# Sexual Practice Changes Post-HIV Diagnosis Among Men Who Have Sex with Men in the United States: A Systematic Review and Meta-analysis

**DOI:** 10.1007/s10461-022-03761-y

**Published:** 2022-07-12

**Authors:** Mohsen Malekinejad, Sopiko Jimsheleishvili, Erin K. Barker, Angela B. Hutchinson, Ram K. Shrestha, Paul Volberding, James G. Kahn

**Affiliations:** 1 Philip R. Lee Institute for Health Policy Studies, University of California, San Francisco, San Francisco, CA USA; 2 Institute for Global Health Sciences, University of California, San Francisco, San Francisco, CA USA; 3 Consortium to Assess Prevention Economics, University of California, San Francisco, San Francisco, CA USA; 4 Division of HIV/AIDS Prevention, Centers for Disease Control and Prevention, Atlanta, GA USA; 5 550 16th Street, San Francisco, CA 94158 USA

**Keywords:** HIV diagnosis, Sero-adaptive behaviors, Systematic review, Men who have sex with men

## Abstract

Men who have sex with men (MSM) often change sexual behaviors following HIV diagnosis. This systematic review examined such changes, including sero-adaptive behaviors (i.e., deliberate safer-sex practices to reduce transmission risk) to better understand the magnitude of their association with HIV diagnosis. We searched four databases (1996–2017) and reviewed references from other systematic reviews. We included studies conducted in the United States that compared sexual behavior among HIV-infected “aware” versus “unaware” MSM. We meta-analytically pooled RRs and associated 95% confidence intervals (CI) using random-effects models, and assessed risk of bias and evidence quality. Twenty studies reported k = 131 effect sizes on sexual practices outcomes, most of which reported changes in unprotected sex (k = 85), and on sex with at-risk partners (k = 76); 11 reported sero-adaptive behaviors. Unprotected anal intercourse with an HIV-uninfected/unknown-status partner was less likely among aware MSM (insertive position: k = 2, RR 0.26, 95% CI 0.17, 0.41; receptive position: k = 2, RR 0.53, 95% CI 0.37, 0.77). Risk of not always serosorting among aware MSM (k = 3) was RR = 0.92 (0.83, 1.02). Existing evidence, although low-quality, suggests that HIV-infected MSM tend to adopt safer sexual practices once aware of their diagnosis. Variation in reporting of outcomes limits their comparability. Sero-adaptive behavior data are sparse.

**Supplementary Information:**

The online version contains supplementary material available at 10.1007/s10461-022-03761-y.

## Introduction

Men who have sex with men (MSM) are the group at greatest risk for HIV infection in the United States (US), comprising more than half of known cases of HIV and an even greater majority (67%) of new HIV infections, according to current surveillance data [1]. Thus, effective HIV prevention strategies targeting MSM are critical to addressing the national burden of HIV. Advances in biomedical HIV prevention strategies stand to protect anyone at risk but in the absence of ideal adherence and efficacy, behavior change remains essential to successful prevention: while uptake of pre-exposure prophylaxis (PrEP) has increased [2], PrEP coverage among MSM at risk of HIV infection is 35–63% [2, 3]. Further, even though antiretroviral therapy (ART) can lead to viral suppression that essentially eliminates transmission risk [4], risk of HIV persists for the many MSM who do not enter treatment right away (28% in 2017) [5] and among those who do not maintain viral suppression [6]. In a recent analysis, only 48.4% of people living with HIV for more than one year in the US were found to have sustained viral suppression [7]. Thus, the ability to make sexual risk decisions based on knowledge of one’s HIV status is a necessary component to interrupting HIV transmission. Similarly, understanding post-diagnosis behavior-change provides critical insight to prevention-strategy planning.

**Fig. 1:**
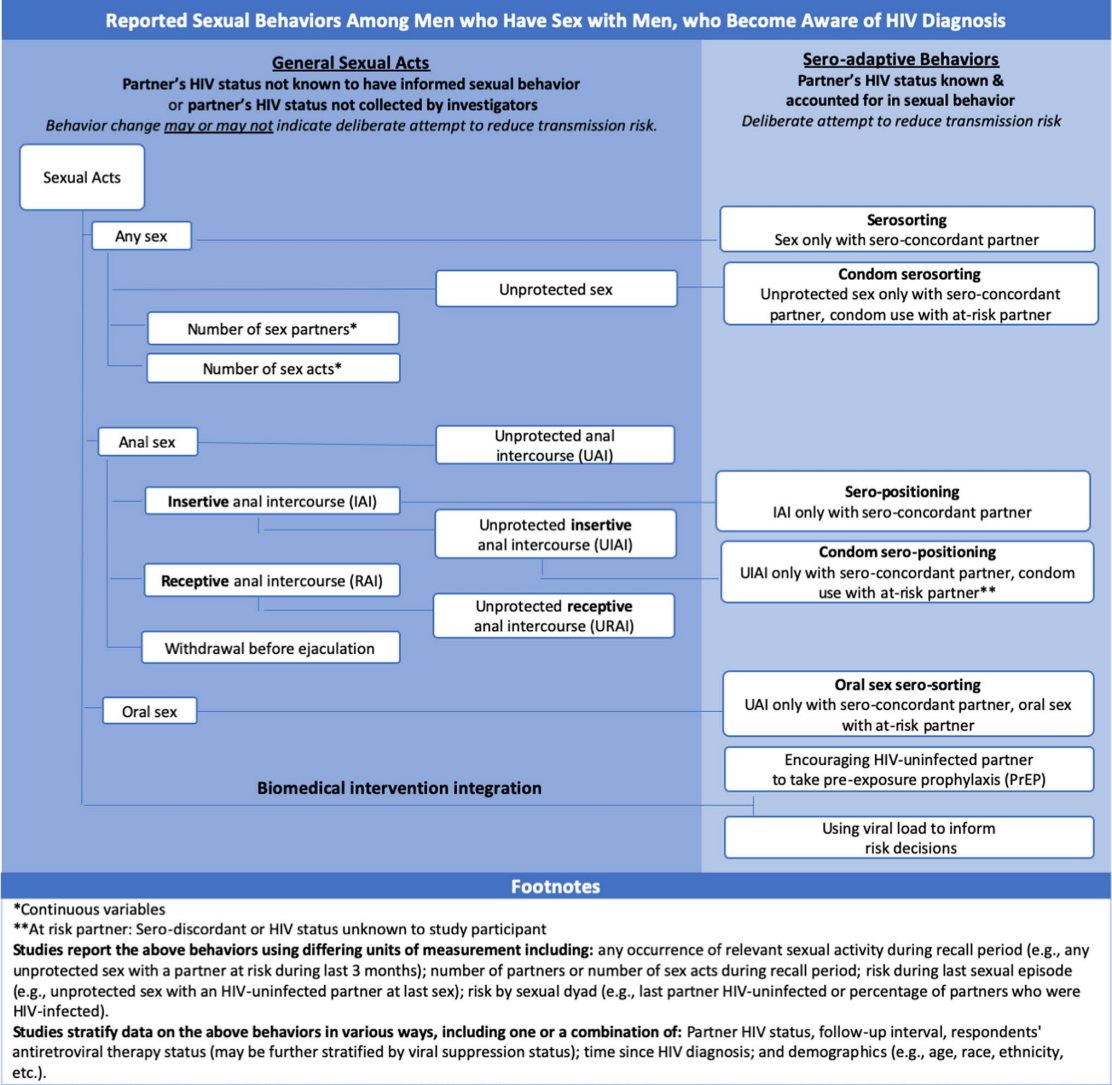
Eligible outcomes for review of changes in sexual practices of men who have sex with men who became aware of HIV diagnosis in the United States

Research has documented a reduction in risky sexual behavior among MSM newly diagnosed with HIV [8, 9]. This includes adopting or increasing standard safer sex practices in general (e.g., condom use, abstinence, etc.) as well as considering a partner’s HIV status when making sexual risk decisions. Some research has examined the practice and community recognition of “sero-adaptive” behaviors among MSM who choose less-risky sexual behaviors with HIV-uninfected partners. This includes “serosorting”—limiting sexual partners to people with concordant HIV status—and “sero-positioning” (also called “strategic positioning”)—where HIV-infected MSM adopt the riskier, receptive role in anal sex with partners who are HIV-uninfected [10–13]. Substantially more research has investigated MSM sexual activity by partner’s HIV status but without considering specifically sero-adaptive practices. All of these research domains contribute to understanding HIV transmission dynamics among MSM, with sero-adaptive categories providing the greatest specificity. Figure 1 illustrates the categorization of and relationships among these various behaviors.

A comprehensive understanding of the effect of HIV diagnosis on sexual risk behavior change must consider sero-adaptive behaviors along with other safer sex practices. Previous systematic reviews have studied serosorting among MSM receiving negative HIV-test results, but not positive results [14, 15]. Other reviews, including one of our own, have examined change in condom use among various groups, including MSM, after receiving an HIV diagnosis [8, 16, 17] but none have specifically analyzed all sero-adaptive and safer-sex behaviors among HIV-infected MSM. Here, we evaluate and present the evidence base for the effect of HIV diagnosis on all sexual risk behaviors among MSM in the US, with meta-analysis where sufficient data were available.

## Methods

This review was based on methods of the Cochrane Collaboration [18]. We developed and followed a protocol we registered in the PROSPERO International Prospective Register of Systematic Reviews (CRD42018085282) [19]. We followed Preferred Reporting Items for Systematic Reviews and Meta-Analyses (PRISMA) guidelines for reporting [20]. We used the Grading of Recommendations Assessment, Development and Evaluation Guideline (GRADE) tool to assess evidence quality by outcome across the literature [21].

### Study Eligibility

We included studies conducted in the US that compared sexual risk behaviors of HIV-infected adult or adolescent MSM aware of their infection versus MSM who were unaware of their infection. Study populations could be of any antiretroviral therapy (ART) status. We excluded studies with data collection completed before 1996, when triple-ART regimens became widely available in the US.

We included any study designs that clearly indicated timing of HIV diagnosis and assessment of outcomes to allow pre- and post-HIV diagnosis comparisons. We included studies that either compared HIV-infected aware MSM vs. HIV-infected but unaware MSM (between-group comparison), or compared HIV-infected MSM before knowledge of diagnosis vs. after receiving diagnosis (within-group comparison). We excluded studies that did not report quantitative epidemiological data (e.g., modeling) or were not rigorously peer-reviewed (e.g., unpublished data, conference proceedings).

Eligible studies could report on ways HIV-infected MSM changed their sexual behavior to account for a partners’ HIV status or on change in sexual behavior overall, which might reflect deliberate efforts to reduce risk but could be due to other factors (e.g., reduced sexual activity due to depression). Figure 1 illustrates eligible outcomes and their interrelationships. Sexual acts included in this review were condom use (overall, for anal sex overall, or for anal sex by role; reported as always using condoms or as the likelihood of using a condom based on condom use reported at last sex), any sex (also reported as abstinence), oral sex, anal sex (overall or by sexual role), withdrawal before ejaculation, number of partners, and number of sex acts.

For the purpose of this review, we defined sero-adaptive behaviors (as opposed to ‘general sexual acts’) when outcomes were measured in a way that could explicitly capture deliberate risk reduction including: serosorting (sex exclusively with sero-concordant partners), seropositioning (anal sex with serodiscordant partners with the HIV-infected partner exclusively in the receptive role), condom serosorting or condom seropositioning (where unprotected sex was restricted as above, but was not necessarily restricted when condoms were used), and oral sex serosorting (where anal sex was restricted as above, but oral sex was not necessarily restricted). We also regarded as sero-adaptive outcomes: (a) the encouragement of an HIV-uninfected partner to take PrEP by an HIV-infected partner or (b) basing sexual decisions on viral load.

### Searches and Screening

We searched PubMed, Embase, PsycInfo, and Web of Science databases using keywords for study design, sexual risk behaviors, MSM populations, and HIV (Supplemental digital content A). The search period for all databases was January 1, 1996–January 15, 2018. We also reviewed references in included papers to identify additional studies. Finally, we included studies from Malekinejad et al. 2021 [16], our recently-completed review on condom use behavior among newly-HIV diagnosed people of any risk group when those studies met the criteria for this review.

**Fig. 2:**
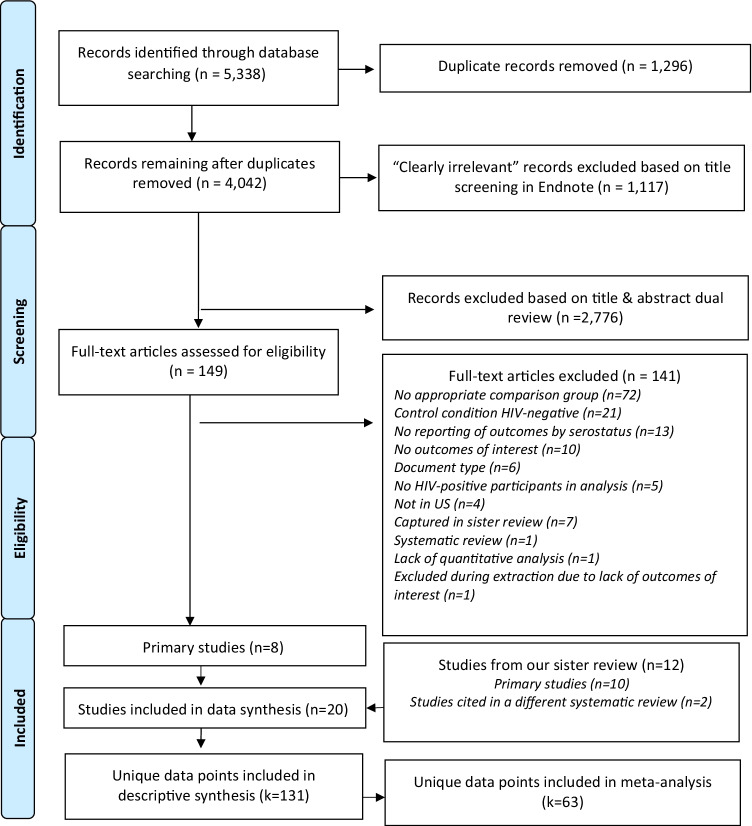
Identification and screening of citations: changes in sexual practices of men who have sex with men who became aware of HIV diagnosis in the United States

We imported all primary records from the database searches into EndNote software version X8 [22]. One reviewer used the search function in EndNote to locate titles with keywords that were likely irrelevant (e.g., “qualitative,” “in-vitro,” see Fig. 2) and reviewed the titles and, if relevant, the abstracts of those records. The two reviewers then independently examined the remaining record titles and, if relevant, abstracts and keywords. The first reviewer also sorted records by study location and excluded studies performed outside of the US. Reviewers resolved incongruent ratings via discussion. They evaluated the full text of included studies independently. A third reviewer was available to resolve disagreements and ambiguities.

### Data Extraction and Standardization

We used a pre-structured data collection spreadsheet to capture the following: study details (e.g., complete citation, geographical setting, study design); descriptions of participants (e.g., age, sex, relevant demographic characteristics); details of HIV testing and comparators (e.g., location and means of HIV testing, HIV testing setting and context, comparison type); outcome definitions and descriptions; details of outcome assessment methods; recruitment methods and eligibility criteria; length of follow-up (time since diagnosis); and data necessary for assessing risk of bias. One reviewer extracted all data, which the second reviewer subsequently reviewed and cross-checked against full-text papers. The two reviewers discussed any disagreements and/or consulted a third rater to adjudicate. When needed, they contacted authors to obtain important missing information from the reports.

### Assessment of Risk of Bias and Quality of Evidence

Two reviewers independently applied criteria recommended by the GRADE Working Group [21] for observational studies: failure of study investigators to develop and apply appropriate eligibility criteria; flawed measurement of exposure and outcome; failure to adequately control confounding; and inadequate follow-up. Each of these assessments helped us determine the quality of evidence for each report and outcome. Raters used a neutral third party to adjudicate disagreements. The quality of evidence was rated as high, unclear, or low based on risk of bias, indirectness, precision, and consistency [21].

### Data Analysis and Synthesis

For dichotomous outcomes, we calculated risk ratios (RR) and 95% confidence intervals (CI). We used the Zhang and Yu method [23] to calculate RR when studies reported odds ratios (OR) for non-rare outcomes. When studies did not report 95% CI, we calculated 95% CI from p-values or from the number of persons with and without the outcome among HIV-infected aware and unaware conditions. For continuous variables, we reported mean difference (MD) and standard deviation (SD) or median and interquartile range (IQR) between the two groups.

To facilitate comparability of findings, we report all RR for undesirable behavior (e.g., unprotected sex instead of condom use). Thus, RR < 1 should be interpreted as decreased risk of undesirable outcomes throughout this review. When we identified two or more contextually compatible outcomes, we performed a meta-analysis using Stata version 14.2 [24]. We used a random-effects model to calculate the pooled effect sizes and 95% CI. We assessed statistical homogeneity using the I^2^ statistic, which reports the overall variation among the pooled data due to heterogeneity, rather than change alone, as a percent value [18]. Because some outcomes combined data on partners who were HIV-uninfected and of unknown HIV status [25], our meta-analyses combine these into a single “partner at risk” group; when studies reported separately on HIV-uninfected and unknown-status partners, we prioritized data for HIV-uninfected partners and report the pooled estimate using data for unknown-status partners in a footnote. Where data were available, we analyzed outcomes for participants on ART. To estimate long-term effects, we pooled data reported for intervals of 12 months or more; if studies reported multiple long-term intervals, we included the longest interval in meta-analysis.

## Results

### Descriptive Analysis

#### Study Screening Results

Our database search returned 4042 unique records, 3893 of which were excluded on the basis of title and/or abstract. We reviewed full text of the remaining 149 articles, and excluded 140 articles that did not meet the inclusion criteria. Of the remaining studies, one was a systematic review that further informed our search. Data from the other eight studies were included in our analysis. We also included data from 12 additional studies reviewed in Malekinejad et al. 2021 [16], resulting in a total of 20 studies. See Fig. 2 and Supplemental digital content B for details.

#### Characteristics of Included Studies

Of the 20 included studies, 13 were cross-sectional and seven had pre-post designs, one of which analyzed and reported data cross-sectionally. Data collection began as early as 1987 [26] and as recently as 2016 [27]. Four studies began prior to 1996. Two studies reported only approximate data collection dates.

Only three studies reported on serosorting [28–30], and one of these studies further differentiated between oral serosorting (serosorting for anal and not oral sex) and condom serosorting (serosorting for unprotected sex but not sex with condoms) [29]. One study reported on seropositioning, which was defined as seropositioning when not using condoms [29].

We reported outcomes by three groups of partner type, depending on the infected person’s knowledge of their partner’s HIV diagnosis. These included: partners who were at risk (n = 11; HIV-uninfected or HIV status not known to the HIV-infected partner), partners whose HIV status was not specified by the authors (n = 18), and partners who were both HIV-infected (n = 2).

Most (n = 13) studies did not report ART uptake among HIV-aware participants. Of those that did, median ART coverage (at follow-up, if reported separately for baseline and follow-up) was 75.8% (IQR 67.0–85.7%). Among prospective cohort studies, follow-up intervals varied from one month to eight years, although the two studies reporting both follow-up intervals and ART coverage had follow-up only at 12 months. Among seven cross-sectional studies that reported on time between HIV diagnosis and time of the sexual behavior in question, the median time interval was nine months (IQR 6.0–12.0 months).

#### Characteristics of Effect Sizes

From 20 studies, we identified a total of k = 131 eligible effect sizes in our analysis (see Table 1 and Supplemental digital content C). We observed great variation in the reported outcomes. Most (k = 86, 66%) effect sizes analyzed risk behavior categorically (e.g., the proportion of participants reporting any occurrence of risk behavior, Supplemental Digital Content C) while the remainder k = 45 reported continuous values (e.g., median number of partners, percentage of condom-protected sex acts, etc.). Seven effect sizes were reported at the sexual dyad level (enumerating partnerships by individual, not sex acts) and twelve reported on last sex or last partner while the remaining k = 112 reported on any outcome of interest during recall periods ranging from 1 to 96 months. Follow-up time was between 6 and 12 months for k = 37 effect sizes (28%), more than 12 months for k = 32 (24%), six months or less for k = 12 (9%), and not reported for k = 50 (38%) effect sizes.

**Table 1:** Characteristics of studies included in review of changes in sexual practices of men who have sex with men who became aware of HIV diagnosis in the United States

Study	Study setting & data collection year	Study design	Participants eligibility criteria	Sample analyzed	Context of comparison by HIV diagnosis status	Outcomes assessed^c^
CDC 2000 [36]^a^	Alabama, New Jersey, and Tennessee 1997–1998	Cross-sectional, participants self-selected for testing	Recently-acquired HIV infection	N = 38	Comparison of aware vs. unaware participants based on officially-reported HIV status	• Any UAI in recall period (duration NR), partner status unspecified: **RR 0.18 (0.09, 0.36)**
CDC 2013 [37]^a^	21 US cities,^i^ 2008 and 2011	Cross-sectional, participants self-selected for testing	Age ≥ 18, male, reporting a male sex partner in the last year, residing in participating city, able to complete questionnaire in English or Spanish	Cycle 2 N = 1558 Unaware: n = 676 White: 17% Age ≥ 30: 82% Cycle 3: N = 1553 Unaware: n = 521 White: 30% Age ≥ 30: 69%	Comparison of aware vs. unaware participants who were tested to validate self-reported HIV status. Cycles were unique samples	• UAI episode with partner at risk in recall period (duration NR) • Cycle 2: **RR 0.53 (0.45, 0.62)** • Cycle 3: **RR 0.45 (0.34, 0.60)**
CDC 2016 [38]^a^	20 US cities,^ii^ 2014	Cross-sectional, participants self-selected for testing	Age ≥ 18, male, reporting oral or anal sex with a male ever, residing in participating city, able to complete survey in English or Spanish	N = 1888	Comparison of aware vs. unaware participants who were tested to validate self-reported HIV status	• Any UAI in recall period (duration NR), partner status unspecified: **RR: 0.43 (0.35, 0.53)** • Any unprotected oral or anal sex in recall period (duration NR), partner status unspecified: RR 1.10 (0.97, 1.25) • Unprotected sex episode with partner at risk during recall period (duration NR): **RR: 0.64 (0.53, 0.77)** • *Number of sex partners in last 12 mo, partner status unspecified:* • *Median (IQR) aware: 4 *[2–10] • *Median (IQR) unaware: 3 *[2–7]
Colfax 2002 [25]^a^	Seven US cities,^iii^ 1995–1999	Single-arm pre-post, participants recruited for testing	Age ≥ 18 males, anal sex with other men in previous year	N = 43 White: 64% Age median: 31 (IQR 20–55) On ART: 70% (at 12 months)	Comparison of the six-month period before first positive HIV test vs. various intervals after diagnosis. Data drawn from a risk-reduction program, with HIV-infected participants offered enrollment in the HIVNET observational cohort	• Any UIAI with partner at risk, 12 mo. post-diagnosis: **RR 0.15 (0.05, 0.49)** • Any UIAI with partner at risk (1 month post-diagnosis): **RR 0.05 (0.01, 0.35)** • *Any UIAI with partner at risk (3 mo. after HIV diagnosis): ****RR 0.21 (0.08, 0.54)*** • Any UIAI with partner at risk (9 mo. post-diagnosis): **RR 0.33 (0.15, 0.75)** • Any UIAI with partner at risk (12 mo. post-diagnosis): **RR 0.31 (0.13, 0.73)** • *Any UIAI with partner at risk (6–12 mo. post diagnosis): ****RR 0.41 (0.19, 0.89)***^*b*^ • Any URAI with partner at risk (1 month post diagnosis): **RR 0.19 (0.08, 0.47)** • *Any URAI with partner at risk (3 mo. after HIV diagnosis): ****RR 0.26 (0.12, 0.56)*** • *Any URAI with partner at risk (6 mo. after HIV diagnosis): ****RR 0.47 (0.26, 0.85)*** • Any URAI with partner at risk (9 mo. post diagnosis): **RR 0.40 (0.21, 0.77)** • Any URAI with partner at risk (12 mo. post diagnosis): **RR 0.30 (0.14, 0.65)**
Darrow 1998 [26]^a^	Miami, Florida 1996	Cross-sectional, participants self-selected for testing	Age ≥ 18 males, unmarried, reporting ever having sex with a man, local resident for at least 30 days	N = 50	Comparison of aware vs. unaware participants based on self-reported HIV status	• Any UAI in last 12 mo., partner status unspecified (median 66 mo. post-diagnosis): RR 2.98 (0.80, 11.17) • Any UAI with partner at risk in last 12 mo. (median 66 mo. post-diagnosis): RR 2.10 (0.61, 7.19)
Drumright 2007 [39]	San Diego and Los Angeles, California 2005	Cross-sectional, participants self-selected for testing	Age ≥ 18 males and females, referred to clinic and completed a computer-assisted self-interview about HIV behaviors	N = 207 individuals reporting on 603 dyads Unaware: n = 144 White: 70.1% Age mean 34; range 18–85	Comparison of before vs. 13 weeks after HIV diagnosis in cross-sectional analysis Participants were referred to the study after diagnosis of recent HIV infection	• Any UAI with any of last 3 sexual partners, 13 weeks post-diagnosis: RR 0.99 (0.77, 1.19)
German 2011 [40]	Baltimore, Maryland Wave 1: 2004–2005 Wave 2: 2008	Cross-sectional, participants self-selected for testing. Two waves with identical protocol, venue-based sampling	Age ≥ 18, male, residents of Baltimore-Townson	N = 243 Unaware: n = 142 White: 11% Age ≥ 25: 84%	Comparison of aware vs. unaware participants who were tested to validate self-reported HIV status	• Any UAI in 12 mo. post-diagnosis, partner status unspecified: • Wave 1: RR 0.94 (0.79, 1.12) • Wave 2: RR 0.97 (0.74, 1.27)
Golden 2008 [34]	Seattle, Washington 2001—2007	Cross-sectional, participants self-selected for testing	MSM seen in the Public Health-Seattle & King County STD Clinic with data allowing classification as serosorter or non-serosorter	N = 1598 Unaware: n = 117 White: 77% Age ≥ 25: 95% *[Unit of analysis is number of visits by MSM]*	Comparison of aware vs. unaware. HIV testing was offered to all participants who had not previously tested positive	• Any AI in 12 mo. post-diagnosis, partner status unspecified: **RR 0.93 (0.92, 0.94)** • Any UAI in 12 mo. post-diagnosis, partner status unspecified: RR 1.03 (0.93, 1.14)
Golden 2012 [28]	Seattle, Washington 2001–2007	Cross-sectional, participants self-selected for testing	MSM seen in the Public Health-Seattle & King County STD Clinic	N = 2763 Unaware: n = 379 White: 79% Age median 40; range 22–46 *[Unit of analysis is number of visits by MSM]*	Comparison of aware vs. unaware. HIV testing was offered to all participants who had not previously tested positive	• Any AI in 12 mo. post-diagnosis, partner status unspecified: **RR 0.97 (0.95, 0.99)** • Any UAI in past 12 mo. post-diagnosis, partner status unspecified: RR 0.97 (0.91, 1.03) • UAI with partner at risk (reported as non-concordant) in 12 mo. post diagnosis: **RR 1.13 (1.03, 1.25)** • Not always serosorting in 12 mo. post-diagnosis: RR 0.98 (0.91, 1.05)
Gorbach 2011 [41]^a^	Southern California 2004—2006	Single-arm pre-post, participants self-selected for testing	People infected with HIV in the last 12 mo	N = 193 White: 71% Age mean: 35 (range 19–64) On ART at every time point in year: 13% 50% were on ART at 9 mo. and at 12 mo	Comparison of before vs after HIV diagnosis. Participants were enrolled in the study following recent HIV diagnosis	• Number of partners in past 3 mo., at the following intervals post-diagnosis: • 3 mo.: **MD − 2.12 (− 4.04, − 0.20)** • 6 mo.: **MD − 2.62 (− 4.48, − 0.76)** • 9 mo.: **MD − 2.67 (− 4.72, − 0.62)** • 12 mo.: **MD − 2.97 (− 5.45, − 0.49)**
Gorbach 2017 [42]	Los Angeles, California 2009–2012	Single-arm pre-post, participants recruited for testing	Age ≥ 18, male, reporting sex with a man in last 12 mo., sought testing at LGBT center	“Recently infected”: N = 125 No demographic data reported “Not recently infected”: N = 90 No demographic data reported	Comparison of before vs. 12 mo. after diagnosis. Participants were enrolled in the study following recent HIV diagnosis. “Recently infected” participants had a negative HIV test in < 12 mo., received diagnosis by physician, shown infection via a NAAT positive test on a negative HIV antibody test, or shown infection via detuned serologic assay. “Not recently infected” had a positive rapid HIV test without any of the above	12 mo. post-diagnosis: • Last partner at risk (irrespective of sexual act or condom use): Recently infected: RR 1.16 (0.83, 1.62) *Not recently infected: RR 1.18 (0.82, 1.69)* • Last sex episode was UIAI with partner at risk*:* *Recently infected: RR 0.83 (0.39, 1.78)* *Not recently infected: RR 1.18 (0.68, 2.02)* • Last sex episode was URAI with partner at risk: Recently infected: RR 0.83 (0.47, 1.45) Not recently infected: RR 0.99 (0.58, 1.68)
Khosropour 2016 [30]^a^	Seattle, Washington 2001–2013	Single-arm pre-post, participants self-selected for testing	Age ≥ 18, male, male sex partner in past 12 mo., visited public health STD clinic at least twice during study period	N = 186 White: 63% Age ≥ 25: 75%	Comparison of before vs. after HIV diagnosis at specified interval. HIV testing was offered to all participants who had not previously tested positive	• UAI with partner at risk (reported as HIV-uninfected partner) at specified interval post-diagnosis: > 1–2 years: **RR 0.17 (0.06, 0.49)** > *2–3 years: ****RR 0.14 (0.05, 0.40)*** > 3–4 years: **RR 0.26 (0.12, 0.58)** • UAI with partner at risk (reported as partner of unknown HIV status) at specified interval post-diagnosis: • > 1–2 years: **RR 0.55 (0.32, 0.94)** • > *2–3 years: ****RR 0.85 (0.52, 1.40)*** • > 3–4 years: **RR 0.36 (0.14, 0.89)** • Not always serosorting at specified interval post-diagnosis: • > 1–2 years: RR 0.93 (0.69, 1.26) • > *2–3 years: RR 0.96 (0.72, 1.28)* • > 3–4 years: RR 0.93 (0.68, 1.28)
Marks 2009 [33]^a^	Los Angeles, California; New York, New York; Philadelphia, Pennsylvania 2005–2006	Cross-sectional, participants self-selected for testing	Age ≥ 18 males, MSM in past 12 mo., Black and Latino	N = 1007 Unaware = 142 White = 0% Median age: Black (43) and Latinos (32) 67% on ART past 3 mon	Comparison of aware vs. unaware participants who were tested to validate self-reported HIV status	Time since diagnosis NR: • Any UAI in past 3 mo., partner status unspecified: **RR 0.75 (0.64, 0.89)** • Any UIAI in past 3 mo., partner status unspecified (on ART): **RR 0.73 (0.59, 0.90)** • Any URAI in past 3 mo., partner status unspecified: RR 0.81 (0.64, 1.01) • UIAI with at-risk partner (reported as HIV-uninfected partner) in past 3 mo.: **RR 0.29 (0.18, 0.46)** • UIAI with at-risk partner (reported as partner of unknown HIV status) in past 3 mo.: **RR 0.45 (0.33, 0.63)** • URAI with partner at risk (reported as HIV-uninfected partner) in past 3 mo.: **RR 0.57 (0.36, 0.91)** • URAI with partner at risk (reported as partner of unknown status) in past 3 mo.: **RR 0.47 (0.33, 0.67)** • UIAI with HIV-infected partner in past 3 mo.: RR 1.51 (1.00, 2.28) • URAI with HIV-infected partner in past 3 mo.: **RR 2.66 (1.60, 4.44)** • UIAI with HIV-infected partner only in past 3 mo.: **RR 2.83 (1.63, 4.91)** • URAI with HIV-infected partner only in past 3 mo.: **RR 3.64 (1.80, 7.35)**
McFarland 2011 [29]^a^	San Francisco Bay Area, California 2007	Cross-sectional, participants self-selected for testing	Age ≥ 18, males, locally resident	N = 262	Comparison of aware vs. unaware participants who were tested to validate self-reported HIV status “Oral sex serosorting” defined as serosorting for anal sex but not oral sex. “Condom serosorting” defined as serosorting for unprotected sex but not sex with condoms. “Condom seropositioning” defined as seropositioning when not using condoms	Individual-level analysis, time since diagnosis NR, recall period of last 6 mo.: • Any sex, partner status unspecified: RR 1.22 (1.0, 1.48) • Any AI, partner status unspecified: RR 1.11 (0.93, 1.32) • Not always using condoms, partner status unspecified: RR 1.18 (0.92, 1.52) • Any AI with a partner at risk: **RR 0.37 (0.23, 0.62)** • All UAI partners serodiscordant: **RR 0.20 (0.07, 0.63)** • Not always serosorting: **RR 0.86 (0.80, 0.93)** • *Not always oral sex serosorting: ****RR 0.97 (0.95, 0.99)*** • *Not always condom serosorting: ****RR 0.94 (0.91, 0.97)*** • *Not always seropositioning: ****RR 0.87 (0.80, 0.94)*** • *Not always condom seropositioning: RR 0.97 (0.91, 1.03)* • *All UAI partners at risk (unknown HIV status): ****RR 89.29 (0.00, 5.91* × *10***^***15***^***)*** • *Partners of mixed serostatus: RR 0.55 (0.27, 1.13)* *Dyad analysis, time since diagnosis NR, recall period of in last 6 mo.:* • *Not always using condoms, partner status unspecified: ****RR 0.87 (0.80, 0.95)*** • *Any AI, partner status unspecified: ****RR 1.19 (1.02, 1.40)*** • *Not always seropositioning: ****RR 0.93 (0.89, 0.97)*** • *Not always condom seropositioning: RR 1.00 (0.98, 1.02)* • *UAI with partner at risk (unknown HIV status): ****RR 0.34 (0.15, 0.74)*** • *Partner serodiscordant: ****RR 0.10 (0.06, 0.18)*** • *UAI partner serodiscordant: ****RR 0.76 (0.70, 0.82)***
McGowan 2004 [43]	Bronx, New York 1997–1998	Cross-sectional, participants self-selected for testing	Age ≥ 18 males and females, documented date of first positive HIV serological test, living in the US for at least 15 years, spoke English, Spanish, French, Twi, Ga, or Hausa	N = 131 Unaware: n = 45 No demographic data reported	Comparison of before vs. after with unspecified follow-up interval. Participants were enrolled in the study following recent HIV diagnosis. Risk reduction counseling was provided throughout the study	• Not always using condoms during recall period (duration NR), partner status unspecified, time since diagnosis NR: **RR 0.42 (0.30, 0.59)**
Saah 1998 (MACS) [44] ^a,b^	4 US cities,^vi^ 1987	Single-arm pre-post, participants recruited for testing	Age ≥ 18, MSM, did not have AIDS at enrollment	N = 90	Comparison of before vs. 18 mo. after HIV diagnosis. No information on testing practices was published	• Any UAI in past 18 mo. since diagnosis: RR 0.75 (0.54, 1.03)
Steward 2009 [32] ^a^	Seven unspecified US cities Year(s) not reported	Single-arm pre-post, participants self-selected for testing	Age ≥ 18 males and females with documented early or acute HIV infection	N = 34 MSM: 93% White: 38% Age mean (SD): 33 (9.5)	Comparison of before vs. after HIV diagnosis at 2 intervals: within 4 weeks of diagnosis (T1) and 8 weeks later (T2). Participants were tested as a part of the study	• Percentage of partners HIV-infected at specified intervals: • *T1: ****RR 0.38 (0.18, 0.79)*** • T2: RR 0.60 (0.36, 1.01) • Last sex episode was unprotected sex: RR 0.77 (0.58, 1.02) • Last sex episode was unprotected sex with HIV-infected partner: • T1: **RR 0.20 (0.08, 0.52)** *• T2: ****RR 0.21 (0.08, 0.53)*** • Number of partners per week, approx. 12 weeks post-diagnosis, partner status unspecified: **MD − 0.73 (− 1.42, − 0.04)**
Vallabhaneni 2013 [27]	San Francisco, California 2016	Single-arm pre-post, participants self-selected for testing	Age ≥ 18, male, acute or recent HIV infection, reporting sex with a male partner in last 3 mo. or self-identified as gay	N = 82 Mean age at diagnosis: 37.5 (SD 8.9)	Comparison of before vs. after HIV diagnosis at specified intervals. Participants were tested as a part of the study	At specified intervals post-diagnosis: • Number of partners in past 3 mo., partner status unspecified: • 6 mo.: **MD − 5.70 (− 11.01, − 0.39)** 12 mo.: **MD − 7.30 (− 12.55, − 2.05)** • 24 mo.: MD − 5.00 (− 10.65, 0.65) • 48 mo.: MD − 2.80 (− 10.19, 4.59) • 60 mo.: MD − 5.00 (− 11.55, 1.55) • 96 mo.: **MD − 7.90 (− 13.20, − 2.60)** • Number of potentially discordant partners in past 3 mo.: • 6 mo.: **MD − 4.80 (− 7.59, − 2.01)** • 12 mo.: **MD − 5.90 (− 8.73, − 3.07)** • 24 mo.: **MD − 4.60 (− 7.53, − 1.67)** 48 mo.: MD − 1.40 (− 6.66, 3.86) • 60 mo.: MD − 2.60 (− 7.76, 2.56) • 96 mo.: **MD − 5.80 (− 8.66, − 2.94)** • Number of potentially discordant partners with whom UAI occurred in past 3 mo.: • 6 mo.: **MD − 2.70 (− 4.72, − 0.68)** • 12 mo.: **MD − 3.30 (− 5.28, − 1.32)** • 24 mo.: **MD − 2.50 (− 4.68, − 0.32)** • 48 mo.: MD − 2.50 (− 5.86, 0.86) • 60 mo.: MD − 3.20 (− 6.70, 0.30) • 96 mo.: **MD − 3.80 (− 7.36, − 0.24)** • Number of potentially discordant partners with whom UIAI occurred: • 6 mo.: MD − 1.80 (− 4.34, 0.74) • 12 mo.: MD − 2.10 (− 4.70, 0.50) • 24 mo.: MD − 2.10 (− 4.77, 0.57) • 48 mo.: MD − 2.20 (− 4.86, 0.46) • 60 mo.: MD − 2.30 (− 4.95, 0.35) • 96 mo.: MD − 2.30 (− 4.93, 0.33) • Number of potentially discordant partners with whom UIAI occurred, controlling for viremia, in past 3 mo.: • 6 mo.: MD − 2.20 (− 4.68, 0.28) • 12 mo.: MD − 2.30 (− 4.81, 0.21) • 24 mo.: MD − 2.30 (− 4.86, 0.26) • 48 mo.: MD − 2.36 (− 4.92, 0.20) • 60 mo.: MD − 2.39 (− 4.93, 0.15) • 96 mo.: MD − 2.39 (− 4.90, 0.12)
Valleroy 2000 [45]	9 US cities, 1994^v^	Cross-sectional, participants self-selected for testing	Residents aged 15–22, sexual experience and orientation were not eligibility criteria	N = 234 34.4% age 15–19 years; 65.6% 20–22	Comparison of aware vs. unaware participants who were tested to validate self-reported HIV status	At least 6 mo. post-diagnosis: • Any UAI in past 6 mo., partner status unspecified: **RR 0.56 (0.32, 0.98)** • Any UIAI in past 6 mo., partner status unspecified: **RR 0.35 (0.14, 0.89)** • Any URAI in past 6 mo., partner status unspecified: RR 0.73 (0.41, 1.30)
Whitham 2018 [46] ^a^	21 unspecified US cities, three rounds of surveys 2008, 2011, 2014	Cross-sectional, participants self-selected for testing	Age ≥ 18 males, ever MSM history	N = 5,079 Aware N = 3,551 On ART N = 2,792 White: 31.3% < age 40: 59.3%	Comparison of aware vs. unaware participants who were tested to validate self-reported HIV status. Sub-analysis by treatment status	All Aware Participants, time since diagnosis NR: • Any UAI with male partner in last 12 mo.: RR 0.46 (0.02, 13.73) • Episode of unprotected sex with at-risk male partner: RR 0.30 (0.00, 122.08) • *Percent reporting* ≥ *10 male AI partners in last 12 mo.: RR 0.62 (0.00, 441.03)* • *Median (IQR) number of male AI partners in last 12 mo.:* *unaware: 3.0 (1–26)* *aware: 3.0 (1–32)* • *Percent reporting* ≥ *10 male UAI partners in last 12 mo.: RR 0.87 (0.00, 13,167.41)* • *Median (IQR) number of male UAI partners in last 12 mo.:* *unaware: 1.0 (0–10)* *aware: 1.0 (0–21)* Aware Participants on ART, time since diagnosis NR: • Any UAI with male partner: **RR 1.11 (1.06, 1.16)** • *Male partner of unknown or HIV-uninfected status at last anal sex: ****RR 0.68 (0.61, 0.77)*** • *Median number of male AI partners in last 12 mo.:* *unaware: 3.0 (1–26)* *aware: 3.0 (1–34)* • *Percent reporting* ≥ *10 male AI partners in last 12 mo.: ****RR 1.52 (1.32, 1.74)*** • *Median number of male UAI partners in last 12 mo.:* *unaware: 1.0 (0–10)* *aware: 1.0 (0–21)* • *Percent reporting* ≥ *10 male UAI partners in last 12 mo.: ****RR 2.02 (1.62, 2.51)***

Bold indicates statistical significance

*Italics* denote data not included in meta-analysis or summary table

“Partner at risk” reflects partners reported as HIV-uninfected and/or with HIV status unknown to the respondent

Cities not specified in table:

^i^Atlanta, Georgia; Baltimore, Maryland; Boston, Massachusetts; Chicago, Illinois; Dallas, Texas; Denver, Colorado; Detroit, Michigan; Houston, Texas; Los Angeles, California; Miami, Florida; New Orleans, Louisiana; Nassau-Suffolk, New York; Newark, New Jersey; New York City, New York; Philadelphia, Pennsylvania; St. Louis, Missouri; San Diego, California; San Francisco, California; San Juan, Puerto Rico; Seattle, Washington; Washington, DC

^ii^Atlanta, Georgia; Baltimore, Maryland; Boston, Massachusetts; Chicago, Illinois; Dallas, Texas; Denver, Colorado; Detroit, Michigan; Houston, Texas; Los Angeles, California; Miami, Florida; New Orleans, Louisiana; Nassau-Suffolk, New York; Newark, New Jersey; New York City, New York; Philadelphia, Pennsylvania; San Diego, California; San Francisco, California; San Juan, Puerto Rico; Seattle, Washington; Washington, DC

^iii^Boston, Massachusetts; Chicago, Illinois; Denver, Colorado; New York, New York; Philadelphia, Pennsylvania; San Francisco, California; Seattle, Washington

^iv^Baltimore, Maryland; Chicago, Illinois; Los Angeles, California; Pittsburgh, Pennsylvania

^v^Baltimore, Maryland; Dallas, Texas; Los Angeles, California; Miami, Florida, New York, New York; San Francisco, Oakland, and San Jose, California; Seattle, Washington

*ART* antiretroviral therapy; *IQR* inter-quartile range; *MD* mean difference; *mo* months; *MSM* men who have sex with men; *RR* risk ratio; *SD* standard deviation; *UAI* unprotected anal intercourse; *UIAI* unprotected insertive anal intercourse; *URAI* unprotected receptive anal intercourse

^a^Identified in a sister systematic review search

^b^Unpublished data reported in Marks 2009 review

^c^Values in parenthesis by effect size: RR (95% confidence interval); median (inter-quartile range); mean difference (confidence interval)

The most commonly reported outcome was UAI, overall (k = 37, 28%) or by role (insertive: k = 26, 20%; receptive: k = 13, 10%). Eleven effect sizes reported sero-adaptive behaviors, five of which were serosorting. Studies also reported on unprotected sex overall (k = 9, 7%) and by number of sexual partners (k = 12, 9%); any anal sex (k = 10, 8%); any sex (k = 1, 1%); and sexual activity by partner HIV status (k = 47, 36%, partner at risk; k = 8, 6%, partner HIV-infected). Seventy-six effect sizes (58%) reported on sex with partners at risk, k = 47 (36%) on partners of unspecified HIV status, and k = 8 (6%) on sex with HIV-infected partners.

### Effects of Being Aware of HIV Diagnosis on Sexual Behaviors

#### When Partners were at Risk

**Fig. 3:**
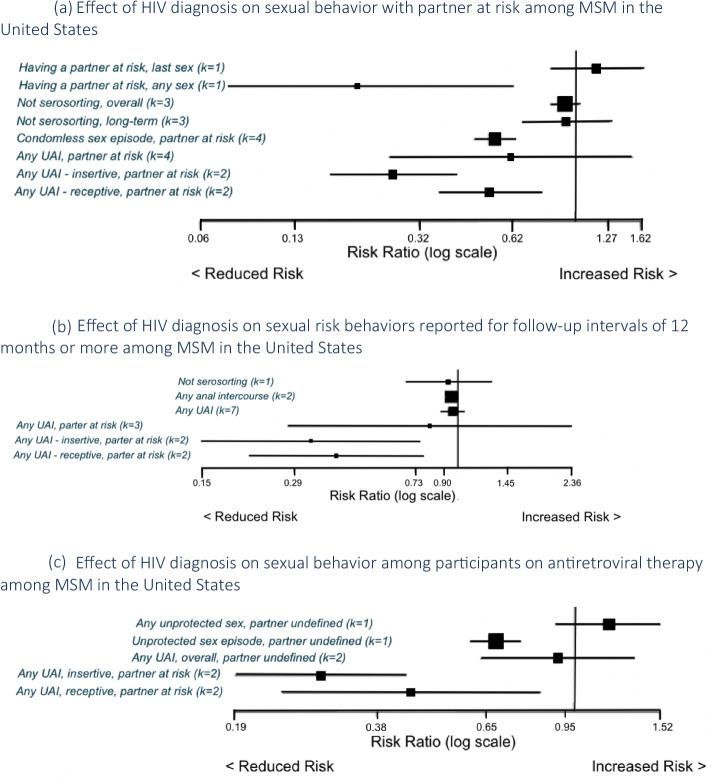
Changes in sexual practices of men who have sex with men who became aware of HIV diagnosis in the United States. **a** Effect of HIV diagnosis on sexual behavior with partner at risk among MSM in the United States. **b** Effect of HIV diagnosis on sexual risk behaviors reported for follow-up intervals of 12 months or more among MSM in the United States. **c** Effect of HIV diagnosis on sexual behavior among participants on antiretroviral therapy among MSM in the United States

Of 76 effect sizes reporting sexual behavior with a partner at risk among aware vs. unaware HIV-infected MSM, we omitted k = 19 from analyses due to overlap. Figure 3a and Table 2 meta-analyzed estimates and unique effect sizes that could not be pooled. The meta-analyzed RR for not serosorting was 0.92 (k = 3, I^2^ = 64%, 95% CI 0.83, 1.02); one study reported an effect size for a long-term effect of 0.93 (95% CI 0.68, 1.28) [31]. The meta-analyzed RR for any unprotected sex episode with a partner at risk was 0.55 (k = 4, I^2^ = 37%, 95% CI 0.47, 0.64) and for any unprotected anal intercourse (UAI) with a partner at risk was 0.62 (k = 4, I^2^ = 91.1%, 95% CI 0.25, 1.52). Specific to sexual role, the meta-analyzed RR for insertive UAI with a partner at risk was 0.26 (k = 2, I^2^ = 0%, 95% CI 0.17, 0.41) and for receptive UAI with a partner at risk was 0.53 (k = 2, I^2^ = 0%, 95% CI 0.37, 0.77). When analysis was restricted to participants on ART, estimates were: insertive RR 0.29 (k = 2, I^2^ = 0%, 95% CI 0.19, 0.44); receptive RR 0.45 (k = 2, I^2^ = 50%, 95% CI 0.24, 0.84, see Fig. 3c).

**Table 2:** Summary of evidence on changes in sexual practices of men who have sex with men who became aware of HIV diagnosis in the United States

Partner at risk (HIV-uninfected or status unknown)							
Outcome	Sub-group analysis	# Data points	Risk ratio or mean difference (95% CI) Pooled if ≥ 2 studies; point estimate or MD range where not pooled	I^2^ (Q test p-value)	Sensitivity Analysis RR Range	GRADE	References (number of data points, if multiple)
Last sexual partner at risk (HIV-uninfected or status unknown)	*Overall*	*1*	*RR 1.16 (0.83, 1.62)*	*n/a*	*n/a*	Very low	Gorbach (2017)
Any sexual partner at risk	*Overall*	*1*	***RR 0.20 (0.07, 0.63)***	*n/a*	*n/a*	Very low	McFarland (2011)
Discordant partnership	*Overall*	*6*	***MD Range [*− *5.90, *− *1.40]***	*n/a*	*n/a*	Very low	Vallabhaneni (2013) (6 f/u time 12–96 mon)
Not always serosorting	Overall	3	RR 0.92 (0.83, 1.02)	64% (0.064)	0.87–0.97 ^a^	Very low	Golden (2012), Khosropour (2016), McFarland (2011)
	*Long-term*	*1*	*RR 0.93 (0.68, 1.28)*	*n/a*	*n/a*	Very low	Khosropour (2016)
Any unprotected anal intercourse	Overall	4	RR 0.62 (0.25, 1.52)^*^	91% (0.000)	0.45–0.87^b^	Very Low	Darrow (1998), Golden (2012), Khosropour (2016), McFarland (2011)
	Long-term	3	RR 0.81 (0.28, 2.36)^†^	86% (0.001)	0.57–1.14^c^	Very Low	Darrow (1998), Golden (2012), Khosropour (2016)
Unprotected sex episode	Overall	4	**RR 0.55 (0.47, 0.64)**	37% (0.188)	0.51–0.57	Low	CDC (2013) (2), CDC (2016) (any sex), Whitham (2018)
	Anal only	3	**RR 0.51 (0.44, 0.58)**	0% (0.589)	0.45–0.53	Low	CDC (2013) (2), Whitham (2018)
	*Anal ART*	*1*	***RR 0.68 (0.61, 0.77)***	*n/a*	*n/a*	Very Low	Whitham (2018)
Any unprotected insertive anal intercourse	Overall	2	**RR 0.26 (0.17, 0.41)**^††^	0% (0.328)	*n/a*	Very Low	Colfax (2002), Marks (2009)
	*Long-term*	*1*	***RR 0.33 (0.15, 0.75)***	*n/a*	*n/a*	Very Low	Colfax (2002)
	*Short-term*	*1*	***RR 0.05 (0.01, 0.35)***	*n/a*	*n/a*	Very low	Colfax (2002)
	On ART	2	**RR 0.29 (0.19, 0.44)**^¶^	0% (0.891)	*n/a*	Low	Colfax (2002), Marks (2009)
Partnership: unprotected insertive anal intercourse—discordant partner	Overall	6	**MD Range [− 2.30, − 1.80]**	n/a	n/a	Very low	Vallabhaneni (2013) (6 f/u time 12–96 mon)
Any unprotected receptive anal intercourse	Overall	2	**RR 0.53 (0.37, 0.77)**^§^	0% (0.593)	*n/a*	Low	Colfax (2002), Marks (2009)
	*Long-term*	*1*	***RR 0.40 (0.21, 0.77)***	*n/a*	*n/a*	Very low	Colfax (2002)
	*Short-term*	*1*	***RR 0.19 (0.08, 0.47)***	*n/a*	*n/a*	Very low	Colfax (2002)
	On ART	2	**RR 0.45 (0.24, 0.84)**^||^	50% (0.158)	*n/a*	Low	Colfax (2002), Marks (2009)
Partnership: unprotected insertive anal intercourse—discordant partner—controlling for viremia	Overall	6	**MD Range [− 2.39, − 2.20]**	n/a	n/a	Very low	Vallabhaneni (2013) (6 f/u time 12–96 mon)

Partner status unspecified							
Outcome	Sub-group analysis	# Data points	Risk ratio or mean difference (95% CI) Pooled if ≥ 2 studies or point estimate [range]	I^2^ (Q test p-value)	Sensitivity analysis RR range	GRADE	References (number of data points, if multiple)
Any sex	*Overall*	*1*	*RR 1.22 (1.00, 1.48)*	*n/a*	*n/a*	Very low	McFarland (2011)
Mean partnerships	Overall	2	**MD − 3.11 (− 5.33, − 0.90)**	13% (0.28)	n/a	Very low	Gorbach (2011), Vallabhaneni (2013)
	Long-term	2	**MD − 4.49 (− 8.54, − 0.44)**	53% (0.14)	n/a	Very low	Gorbach (2011), Vallabhaneni (2013)
	*Any partners*	*6*	***MD range [*− *7.90, *− *2.80]***	*n/a*	*n/a*	Very low	Vallabhaneni (2013) (6 f/u time 12–96 mon)
	Last 3 months	4	**MD − 2.55 (− 3.57, − 1.53)**	0% (0.96)	n/a	Very low	Gorbach (2011) (4)
	*Last week*	*1*	***MD *− *0.73 (*− *1.42, *− *0.04)***	*n/a*	*n/a*	Very low	Steward (2009)
Any anal sex	Overall	3	RR 0.96 (0.92, 1.00)	86% (0.001)	0.95–1.01^d^	Very low	Golden (2008), Golden (2012), McFarland (2011)
	Long-term	2	**RR 0.95 (0.91, 0.99)**	91% (0.001)	0.93–0.97^e^	Very low	Golden (2008, 2012)
Episode of unprotected sex	Overall	2	RR 0.88 (0.69, 1.12)	42% (0.188)	0.77–0.99^f^	Very low	Drumright (2007), Steward (2009)
	*Anal only*	*1*	*RR 0.99 (0.77, 1.19)*	*n/a*	*n/a*	Very low	Drumright (2007)
Any unprotected sex—any sex type	Overall	3	RR 0.84 (0.51, 1.38)	93% (0.000)	0.69–1.11^ g^	Very low	CDC (2016), McFarland (2011), McGowan (2004)
	*ART*	*1*	*RR 1.18 (0.92, 1.52)*	*n/a*	*n/a*	Very low	McFarland (2011)
Any unprotected anal intercourse	Overall	11	**RR 0.75 (0.62, 0.91)**	89% (0.000)	0.75–0.84^h^	Very low	CDC (2000, 2016), Darrow (1998), German (2011) (2), Golden (2008), Golden (2012), Marks (2009), Saah (1998), Valleroy (2000), Whitham (2018)
	Long-term	7	RR 0.96 (0.87, 1.05)	41% (0.118)	0.92–0.97	Low	Darrow (1998), German (2011) (2), Golden (2008, 2012), Saah (1998), Valleroy (2000)
	ART	2	RR 0.92 (0.63, 1.34)	95% (0.000)	0.75–1.11^i^	Very low	Marks (2009), Whitham (2018)
Any unprotected insertive anal intercourse	Overall	2	RR 0.58 (0.30, 1.12)	56% (0.130)	0.35–0.73^j^	Very low	Marks (2009), Valleroy (2000)
Unprotected sex episode – insertive anal intercourse	*Overall*	*2*	*RR 0.83 (0.39, 1.78)*	*n/a*	*n/a*		Gorbach (2017) (2 samples: recently infected, not recently infected)
			*RR 1.18 (0.68, 2.02)*			Very low	
Any unprotected receptive anal intercourse	Overall	2	**RR 0.79 (0.64, 0.98)**	0%, 0.736	0.73–0.81^ k^	Very low	Marks (2009), Valleroy (2000)
Episode of unprotected receptive anal intercourse	*Overall*	*2*	*RR 0.83 (0.47, 1.45)*	*n/a*	*n/a*		Gorbach (2017) (2 samples: recently infected, not recently infected)
			*RR 0.99 (0.58, 1.68*			Very Low	

Partner HIV-infected							
Outcome	Sub-group analysis	# Data points	Risk ratio or mean difference (95% CI) pooled if ≥ 2 studies	I^2^ (Q test p-value)	Sensitivity analysis RR range	GRADE	References (number of data points, if multiple)
Percent of sexual partners who were HIV-infected	*Overall*	*1*	*RR 0.60 (0.36, 1.01)*	*n/a*	*n/a*	Very Low	Steward (2009)
Episode of unprotected sex—HIV-infected partner	*Overall*	*1*	***RR 0.21 (0.08, 0.53)***	*n/a*	*n/a*	Very Low	Steward (2009)
Any unprotected insertive anal intercourse—HIV-infected partner	*Overall*	*1*	*RR 1.51 (1.00, 2.28)*	*n/a*	*n/a*	Very Low	Marks (2009)
Any unprotected receptive anal sex—HIV-infected partner	*Overall*	*1*	***RR 2.66 (1.60, 4.44)***	*n/a*	*n/a*	Very Low	Marks (2009)

Bolding indicates statistical significance. *Italics* indicate estimates not meta-analyzed

*ART* antiretroviral therapy, *Long-term* longest follow-up period available within each study, *n/a* not applicable, *RR* risk ratio, *Short-term* shortest follow-up period available within studies

^*^Reflects data from Khosropour 2016 on partners known to be HIV-uninfected; when partners of unknown HIV status is used, RR = 0.76 (I^2^ = 89%, p = 0.000; 95% CI 0.38, 1.52)

^†^Reflects data from Khosropour 2016 on partners known to be HIV-uninfected; when partners of unknown HIV status is used, RR = 0.93 (I^2^ = 70%, p = 0.037, 95% CI 0.42, 2.08)

^††^Reflects data from Marks 2009 on partners known to be HIV-uninfected; when partners of unknown HIV status is used, RR = 0.31 ((I^2^ = 68%, p = 0.079, 95% CI 0.11, 0.85)

^¶^Reflects data from Marks 2009 on partners known to be HIV-uninfected; when partners of unknown HIV status is used, RR = 0.43 ((I^2^ = 0%, p = 0.411, 95% CI 0.19, 0.44)

^§^Reflects data from Marks 2009 on partners known to be HIV-uninfected; when partners of unknown HIV status is used, RR = 0.47 (I^2^ = 0%, p = 0.973, 95% CI 0.35, 0.64)

^||^Reflects data from Marks 2009 on partners known to be HIV-uninfected; when partners of unknown HIV status is used, RR = 0.43 (I^2^ = 11%, p = 0.289, 95% CI 0.30, 0.62)

Sensitivity Analyses (where removal of one data point results in a change of effect size ≥ 0.05):

^a^0.87 (0.80, 0.93) without Golden 2012, 0.97 (0.91, 1.04) without McFarland 2011

^b^0.45 (0.16, 1.31) without Darrow 1998, 0.47 (0.15, 1.48) without Golden 2012, 0.87 (0.35, 2.14) without Khosropour 2016, 0.75 (0.22, 2.55) without McFarland 2011

^c^0.57 (0.14, 2.41) without Darrow 1998, 0.70 (0.09, 5.40) without Golden 2012, 1.14 (1.03, 1.25) without one Khosropour 2016

^e^1.01 (0.90, 1.13) without Golden 2008

^f^0.97 (0.95, 0.99) without Golden 2008, 0.93 (0.92, 0.94) without Golden 2012

^g^0.77 (0.58, 1.01) without Drumright 2007, 0.99 (0.77, 1.27) without Steward 2009

^h^0.71 (0.26, 1.95) without CDC 2016, 0.69 (0.27, 1.77) without McFarland 2011, 1.11 (1.00, 1.24) without McGowan 2004

^i^0.81(0.68, 0.97) without CDC 2000, 0.84 (0.73, 0.98) without CDC 2016

^j^1.11 (1.06, 1.16) without Marks 2009, 0.75 (0.64, 0.89) without Whitham 2018

^k^0.35 (0.14, 0.88) without Marks 2009, 0.73 (0.59, 0.90) without Valleroy 2000

^l^0.73 (0.41, 1.28) without Marks 2009

When data were restricted to follow-up intervals of 12 months or more, results for UAI with a partner at risk were RR 0.81, k = 3, I^2^ = 86%, 95% CI 0.28, 2.36 (see Fig. 3b). One study reported mean differences for having a discordant partnership as ranging from − 5.90 to − 1.40 over follow-up intervals of 12 to 96 months [27].

#### When Partners were HIV-Infected

Four different outcomes where both partners were HIV-infected were reported across four studies. The RR reported for the percentage of partners who were HIV infected before and after diagnosis was 0.60 (k = 1, 95% CI 0.36, 1.01) and for an episode of unprotected sex was 0.21 (k = 1, 95% CI 0.08, 0.53) [32]. The RR for any insertive UAI with an HIV-infected partner among aware vs. unaware MSM was 1.51 (k = 1, 95% CI 1.00, 2.28) and 2.66 (k = 1, 95% CI 1.60, 4.44) for receptive UAI with an HIV-infected partner [33].

#### When Partners’ HIV Status was not Specified

Eighteen studies reported k = 47 effect sizes for sexual behavior with a partner of unspecified HIV status. The meta-analyzed mean difference in number of partnerships was − 3.11 (k = 2, I^2^ = 13%, 95% CI − 5.33, − 0.90). Meta-analyzed RR for any anal sex was 0.96 (k = 3, I^2^ = 86%, 95% CI 0.92, 1.00) and for any UAI as 0.75 (k = 11, I^2^ = 89%, 95% CI 0.62, 0.91), and found a reduction in receptive UAI (RR 0.79, k = 2, I^2^ = 0%, 95% CI 0.64, 0.98). Long-term effects (follow-up intervals of 12 months or more) for any anal sex were RR 0.95 (k = 2, I^2^ = 91%, 95% CI 0.91, 0.99) and for any UAI were RR 0.96 (k = 2, I^2^ = 41%, 95% CI 0.87, 1.05, see Fig. 3b). When analysis was restricted to participants on ART, results for any UAI were RR 0.92 (k = 2, I^2^ = 95%, 95% CI 0.63, 1.34, see Fig. 3c).

### Risk of Bias

We identified four study designs, described previously [16]. Studies included in this review included two kinds of within-group comparison pre-post designs, where participants were either recruited (n = 2) or self-selected (n = 5) for testing, as well as cross-sectional studies where participants were self-selected for testing (n = 13). See Supplemental digital content D.

Risk of bias was generally high or unclear, largely due to the observational nature of the studies and the need to rely on self-reported data. All studies were rated at high risk of bias in the category of measurement of exposure and/or outcome, due to self-reported outcomes. Additionally, most effect sizes were rated at high or unclear risk of bias in data completion (including description of exclusion criteria and attrition, 104, 79%), selective outcome reporting (89, 68%), control for confounders (89, 68%), and other unspecified bias (116, 89%). Low risk of bias was identified for all effect sizes regarding development and application of eligibility criteria, and for all but two effect sizes in follow-up time, which were unclear. (See Supplemental digital content E and F.)

## Discussion

To our knowledge, this review is the first of its kind addressing the association between HIV diagnosis knowledge and a wide range of sexual practices. Prior reviews [14, 15] have examined behavior change among HIV-uninfected MSM who engage in serosorting to protect themselves from HIV infection, but not focused on MSM receiving HIV diagnoses whose behavior change intends to prevent onward transmission. We found limited data on serosorting and seropositioning, and more data on condom use, sexual activity and discordant partnerships. This review provides an assessment of sero-adaptive and other sexual risk behavior change among HIV-infected MSM after learning of their diagnosis.

HIV-infected aware MSM have a lower risk of UAI with partners at risk, compared to those not aware of their infection (RR = 0.62). The reduction in UAI risk was observed for both receptive (RR = 0.53) and insertive (RR = 0.26) anal sex. This suggests that knowledge of positive HIV status was an important factor in reducing UAI and, therefore, expanded HIV testing may result in lower risk behavior among MSM who would otherwise be unaware of their infection. MSM aware of their HIV infection are also less likely to report sex with partners at risk, irrespective of condom use and fewer sexual partners.

HIV-infected-aware participants on ART had a lower risk of engaging in UAI with partners at risk and with partners whose HIV status was unspecified in studies. This held true at long-term follow-up of 12 months or more, though neither of these findings were statistically significant.

This review found somewhat different results for UAI than our prior review of condom use among various risk groups [16]. For example, the prior review estimated the RR for UAI with a partner at risk among aware MSM as 0.46 (k = 6, I^2^ = 60%, 95% CI 0.30, 0.70) while this review estimated an RR of 0.62 (k = 4, I^2^ = 90%, 95% CI 0.26, 1.52). We attribute such differences to changes in eligibility criteria: specifically, the prior review included data collected in Canada as well as conference abstracts, whereas this review was restricted to peer-reviewed studies conducted in the US. Notably, this review searched for keywords and captured outcomes beyond condom use which, although seldom reported, provide additional context and detail for the MSM population.

Limitations of this review reflect the limitations of primary studies, primarily the limited body of evidence on sero-adaptive behaviors; also bias due to self-reported behavioral outcomes. Additionally, the variation in how sexual behavior was reported by primary studies limited the volume of data available for pooled estimates. For example, “serosorting” was defined in different studies as: any sex exclusively with sero-concordant partners [29]; UAI only with sero-concordant partners [28, 34]; or not using condoms with a partner of concordant HIV status (with no discussion of serodiscordant sexual contact) [35]. Within these definitions, “concordant” may refer either to actual HIV status at or before HIV diagnosis (i.e., sex with an HIV-infected partner [28, 34]) or perceived HIV status (i.e., sex with an HIV-uninfected partner [31]). Additionally, where general sexual acts were reported, it is possible that reported sexual activity and condom use reflected some sero-adaptive behavior (e.g., reports of any receptive anal intercourse (RAI) could include participants who engaged in seropositioning), but this cannot be known definitively. Similarly, data on any anal sex and any oral sex could capture participants who switched from anal sex to oral sex to reduce risk or those who engaged in oral sex serosorting.

Such complexity poses a challenge to understanding what effect HIV diagnosis truly has on risk behavior among MSM. In meta-analysis, we combined data with equivalent operational definitions. While transforming outcome data to RR facilitated comparability of findings across studies, many included studies are cross-sectional in design with limitations for developing a true estimate of risk. Further, given the diversity of the outcome types, it was prudent to apply a random-effects model to account for source of heterogeneity.

Finally, because few studies stratified results by participants’ ART status and no studies addressed PrEP use by HIV-uninfected partners, these factors are not fully addressed in our review.

Future research should attend to the above nuances and associated contextual factors in order to expand the body of evidence on sero-adaptive behaviors among MSM. In particular, studies should attempt to address pure serosorting and seropositioning behaviors—i.e., the degree to which UAI or insertive UAI is practiced with HIV-concordant partners at the exclusion of discordant partners. While existing data on “any” occurrence of sex with partners of each serostatus is helpful to both mathematical modeling and HIV prevention efforts, addressing the holistic context of participants’ sexual behavior through comprehensive data collection and analysis could further enhance HIV prevention strategies.

## Supplementary Information

Below is the link to the electronic supplementary material.

Supplementary file1 (DOCX 4325 KB)

## Data Availability

Upon request.
