# Supplementary material for: Sexual Practice Changes Post-HIV Diagnosis Among Men Who Have Sex with Men in the United States: A Systematic Review and Meta-analysis
Source: AIDS Behav. 2022 Jul 12;27(1):257–78. doi: 10.1007/s10461-022-03761-y (PMC9834435; doi:10.1007/s10461-022-03761-y)
Supplement: Supplementary file 1 — Supplementary file1 (DOCX 4325 KB) [file 10461_2022_3761_MOESM1_ESM.docx]

**Sexual practice changes post-HIV diagnosis among men who have sex with men in the United States: A systematic review and meta-analysis**

**Supplemental digital content**

[Supplemental digital content A: Electronic database search strategies on changes in sexual practices of men who have sex with men who became aware of HIV diagnosis in the United States (9 January 2018) and first cut of titles (15 January 2018) 2](#_Toc57898360)

[Supplemental digital content B: Articles screened at the full text level on changes in sexual practices of men who have sex with men who became aware of HIV diagnosis in the United States 17](#_Toc57898361)

[Supplemental digital content C: Summary of characteristics of studies and effect sizes reporting on changes in sexual practices of men who have sex with men who became aware of HIV diagnosis in the United States 37](#_Toc57898362)

[Supplemental digital content D: Study designs and important risk of bias for included studies on changes in sexual practices of men who have sex with men who became aware of HIV diagnosis in the United States 39](#_Toc57898363)

[Supplemental digital content E: Risk of bias among included studies on changes in sexual practices of men who have sex with men who became aware of HIV diagnosis in the United States 40](#_Toc57898364)

[Supplemental digital content F: Risk of bias of included studies on changes in sexual practices of men who have sex with men who became aware of HIV diagnosis in the United States 41](#_Toc57898365)

## Supplemental digital content A: Electronic database search strategies on changes in sexual practices of men who have sex with men who became aware of HIV diagnosis in the United States (9 January 2018) and first cut of titles (15 January 2018)

**Contents**

1. Database Searches
   1. Yield of databases
   2. PubMed search strategy
   3. Embase search strategy
   4. Web of Science search strategy
   5. De-duplication process in EndNote
2. First Cut Using Title Search in EndNote
   1. Summary of de-duplication and “first cut” of clearly irrelevant records*
   2. Title keyword searches within EndNote

***************************

1. **Database Searches: Sero-adaptive behavior among MSM who know they have HIV infection**
2. **Yield of databases**

| **PubMed** | **1,594** |
| --- | --- |
| **Embase** | **2,656** |
| **PsycInfo** | **1,088** |
| **­­Gross total** | **5,338** |
| **Auto-identified duplicates** | **992** |
| **NET TOTAL** | **4,346** |

1. **PubMed search strategy:**

| **Search** | **PubMed Query** | **Items found 9 Jan. ‘18** |
| --- | --- | --- |
| #7 | #6 AND date limit 1996-01-01 to 2018-01-09 | 1,594 |
| #6 | #1 AND #2 AND #3 AND #4 AND #5 | 1,962 |
| #5 | randomized controlled trial[pt] OR controlled clinical trial[pt] OR randomized controlled trials[mh] OR random allocation[mh] OR random*[tiab] OR trial*[tiab] OR prospective studies[mh] OR retrospective[tiab] OR intervention*[tiab] OR case-control[tiab] OR "Cross-Sectional Studies"[mh] OR cross-section*[tiab] OR observational[tiab] OR cohort studies[mh] OR program evaluation[mh] OR non-random*[tiab] OR nonrandom*[tiab] OR “before and after”[tiab] OR “time series”[tiab] OR cohort*[tiab] OR intervention*[tiab] OR prospective*[tiab] OR longitud*[tiab] OR (pre[tiab] AND post[tiab]) OR evaluation[tiab] OR "Time factors"[mh] OR “National HIV Behavioral Surveillance”[tiab] | 6,009,070 |
| #4 | “HIV Serosorting”[mh] OR “HIV Seropositivity”[mh] OR “Sexual Partners”[mh] OR “Sexual Behavior”[mh] OR (serostatus[tiab] AND knowledge[tiab]) OR (serostatus[tiab] AND behav*[tiab]) OR (serostatus[tiab] AND partner*[tiab]) OR (“sero-status”[tiab] AND knowledge[tiab]) OR (“sero-status”[tiab] AND behav*[tiab]) OR (“sero-status”[tiab] AND partner*[tiab]) OR serodiscordan*[tiab] OR sero-discordan*[tiab] OR HIV-discordan*[tiab] OR discordan*[tiab] OR serosort*[tiab] OR sero-sort*[tiab] OR seroadapt*[tiab] OR sero-adapt*[tiab] OR seroposition*[tiab] OR sero-position*[tiab] OR serocommunication[tiab] OR sero-communication[tiab] OR “partner selection”[tiab] OR “partner choice”[tiab] OR “sexual harm reduction”[tiab] OR “strategic positioning”[tiab] OR seroguess*[tiab] OR sero-guess*[tiab] OR "sexual abstinence"[mh] OR abstinen*[tiab] OR celiba*[tiab] OR “safe sex”[mh] OR “safe* sex”[tiab] OR “Pre-Exposure Prophylaxis” OR PrEP[tiab] OR withdraw*[tiab] OR “viral load”[tiab] OR "Risk-Taking"[mh] OR "risk taking"[tiab] OR "risk-taking"[tiab] OR "Risk Reduction Behavior"[mh] OR "Unsafe Sex/statistics & numerical data"[mh] | 327,301 |
| #3 | “HIV Infections/Diagnosis”[mh] OR "AIDS serodiagnosis"[mh] OR "rapid test*"[tiab] OR "rapid HIV test*"[tiab] OR diagnosed[tiab] OR diagnosis[tiab] OR “test results”[tiab] OR "received results"[tiab] OR "received a result" OR "received positive"[tiab] OR "tested positive"[tiab] OR "test positive" OR "HIV counsel*"[tiab] OR "HIV test*"[tiab] OR HCT[tiab] OR VCT[tiab] OR "Mass Screening/psychology"[mh] OR "community health services/Counseling"[mh] | 1,765,181 |
| #2 | “Homosexuality, Male/psychology”[mh] OR homosexual*[tiab] OR Bisexuality/psychology[mh] OR bisexual*[tiab] OR gay[tiab] OR gays[tiab] OR “men who have sex with men”[tiab] OR MSM[tiab] OR transgender[tiab] OR transsexual*[tiab] OR transwomen[tiab] OR transvestite*[tiab] | 35,657 |
| #1 | HIV Infections[mh] OR HIV*[mh] OR HIV[tiab] OR HIV/AIDS[tiab] OR hiv-1[tiab] OR hiv1[tiab] OR human immunodeficiency virus[tiab] OR human immune deficiency virus[tiab] OR human immuno-deficiency virus[tiab] OR human immune-deficiency virus[tiab] OR ((human immun*) AND (deficiency virus[tiab])) OR acquired immunodeficiency syndromes[tiab] OR acquired immune deficiency syndrome[tiab] OR acquired immuno-deficiency syndrome[tiab] OR acquired immunedeficiency syndrome[tiab] OR ((acquired immun*) AND (deficiency syndrome[tiab])) | 366,209 |

**3. Embase search strategy**

| **Search** | **EMASE Query** | **Items found 9 Jan. ‘18** |
| --- | --- | --- |
| #8 | #6 NOT 'conference abstract'/it | 2,656 |
| #7 | #6 NOT [10-1-2018]/sd AND [1996-2018]/py (completed 1996-2018, added to EMBASE before 10 January 2018) | 3,670 |
| #6 | #1 AND #2 AND #3 AND #4 AND #5 | 3,898 |
| #5 | 'hiv test'/exp OR 'hiv test*':ti OR 'hiv test*':ab OR 'rapid test'/exp OR 'hiv serodiagnosis'/exp OR 'rapid hiv test*':ti OR 'rapid hiv test*':ab OR (hiv:ti AND (diagnos*:ti OR serodiagnosis:ti)) OR (hiv:ti AND (status:ti OR serostatus:ti) AND know*:ti) OR (hiv:ab AND (diagnos*:ab OR serodiagnosis:ab)) OR (hiv:ab AND (status:ab OR serostatus:ab) AND know*:ab) OR 'test result*':ti OR 'test result*':ab OR 'received results':ti OR 'received results':ab OR 'received a result':ti OR 'received a result':ab OR 'positive result*':ti OR 'positive result*':ab OR 'test positive':ti OR 'test positive':ab OR 'tested positive':ti OR 'tested positive':ab OR 'hiv counsel*':ti OR 'hiv counsel':ab OR hct:ti OR hct:ab OR vct:ti OR vct:ab OR (hiv:ti AND screen*:ti) OR (hiv:ab AND screen*:ab) OR (hiv:ti AND counsel*:ti) OR (hiv:ab AND counsel*:ab) OR 'mass screening'/exp/mj OR 'anonymous testing'/exp | 361,257 |
| #4 | 'hiv serosorting'/exp OR 'sexual partner*':ti OR 'sexual partner*':ab OR 'sexual behavior'/exp OR 'sexual behav*':ti OR 'sexual behav*':ab OR ((serostatus:ti OR 'sero-status':ti) AND (know*:ti OR behav*:ti OR partner*:ti)) OR ((serostatus:ab OR 'sero-status':ab) AND (know*:ab OR behav*:ab OR partner*:ab)) OR 'serodiscordant couple'/exp OR serodiscord*:ti OR serodiscord*:ab OR 'sero discord*':ti OR 'sero discord*':ab OR 'sero-discord*':ti OR 'sero-discord*':ab OR 'hiv discord*':ti OR 'hiv discord*':ab OR 'hiv-discord*':ti OR 'hiv-discord*':ab OR serosort*:ti OR serosort*:ab OR 'sero sort*':ti OR 'sero sort*':ab OR 'sero-sort*':ti OR 'sero-sort*':ab OR seroadapt*:ti OR seroadapt*:ab OR 'sero adapt*':ti OR 'sero adapt*':ab OR 'sero-adapt*':ti OR 'sero-adapt*':ab OR seroposition*:ti OR seroposition*:ab OR 'sero position*':ti OR 'sero position*':ab OR 'sero-position*':ti OR 'sero-position*':ab OR serocommunication:ti OR serocommunication:ab OR 'sero-communication':ti OR 'sero-communication':ab OR 'partner selection':ti OR 'partner selection':ab OR 'partner choice':ti OR 'partner choice':ab OR 'sexual harm reduction':ti OR 'sexual harm reduction':ab OR 'strategic positioning':ti OR 'strategic positioning':ab OR seroguess*:ti OR seroguess*ab OR 'sero guess*':ti OR 'sero guess*':ab OR 'sero-guess':ti OR 'sero-guess':ab OR 'sexual abstinence'/exp OR abstinence:ti OR abstinence:ab OR abstain*:ti OR abstain*:ab OR celiba*:ti OR celiba*:ab OR 'safe sex'/exp OR 'safe sex':ti OR 'safe sex':ab OR 'safer sex':ti OR 'safer sex':ab OR 'unsafe sex'/exp OR 'unsafe sex':ti OR 'unsafe sex':ab OR (partner*:ti AND ('pre-exposure prophylaxis'/exp OR prep:ti OR withdraw*:ti OR 'viral load':ti)) OR (partner*:ab AND ('pre-exposure prophylaxis'/exp OR prep:ab OR withdraw*:ab OR 'viral load':ab)) OR 'high risk behavior'/exp OR 'risk behavior*':ti OR 'risk behavior*':ab OR 'risk taking':ti OR 'risk taking':ab OR 'risk-taking':ti OR 'risk-taking':ab OR 'risk reduction':ti OR 'risk reduction':ab | 270,666 |
| #3 | 'homosexual male'/exp OR 'bisexual male'/exp OR homosex*:ti OR homosex*:ab OR bisex*:ti OR bisex*:ab OR gay*:ti OR gay*:ab OR 'men who have sex with men'/exp OR 'men who have sex with men and women'/exp OR 'men who have sex with men':ti OR 'men who have sex with men':ab OR msm:ti OR msm:ab OR 'transgender'/exp OR transgender*:ti OR transgender*:ab OR transsexual*:ti OR transsexual*:ab OR transvestite*:ti OR transvestite*:ab | 44,285 |
| #2 | 'statistical analysis'/exp OR 'statistical analysis' OR risk:ti OR risk:ab OR controlled:ti OR controlled:ab OR predictor*:ti OR predictor*:ab OR correlate*:ti OR correlate*:ab OR determinant*:ti OR determinant*:ab OR cofactor*:ti OR cofactor*:ab OR 'co-factor':ti OR 'co-factor':ab OR 'risk factor':ti OR 'risk factor':ab OR 'randomized controlled trial'/exp OR 'randomized controlled trial' OR random*:ti OR random*:ab OR trial*:ti OR trial*:ab OR 'prospective study'/exp OR 'prospective study' OR 'retrospective study'/exp OR 'retrospective study' OR prospective:ti OR prospective:ab OR retrospective:ti OR retrospective:ab OR 'intervention study'/exp OR 'intervention study' OR intervention*:ti OR intervention:ab OR 'case control study'/exp OR 'case control study' OR 'case control':ti OR 'case control':ab OR 'cross-sectional study'/exp OR 'cross-sectional study' OR 'cross sectional':ti OR 'cross sectional':ab OR 'cross-sectional':ti OR 'cross-sectional':ab OR 'observational study'/exp OR 'observational study' OR observational:ti OR observational:ab OR 'cohort analysis'/exp OR 'cohort analysis' OR cohort*:ti OR cohort*:ab OR 'evaluation study'/exp OR 'evaluation study' OR evaluat*:ti OR evaluat*:ab OR 'non-random':ti OR 'non-random':ab OR 'before and after':ti OR 'before and after':ab OR 'time series':ti OR 'time series':ab OR 'time series analysis'/exp OR 'time series analysis' OR 'longitudinal study'/exp OR 'longitudinal study' OR longitudinal*:ti OR longitudinal*:ab OR (pre:ti AND post:ti) OR (pre:ab AND post:ab) OR 'time factor':ti OR 'time factor':ab OR 'national hiv behavioral surveillance':ti OR 'national hiv behavioral surveillance':ab | 10,396,215 |
| #1 | 'human immunodeficiency virus infection'/exp/mj OR 'human immunodeficiency virus infection' OR 'human immunodeficiency':ti OR 'human immunodeficiency':ab OR hiv*:ti OR hiv*:ab | 473,738 |

**4. PsycInfo search strategy**

| **Search** | **PsycInfo Query** | **Items found 9 Jan. ‘18** |
| --- | --- | --- |
|  | Unique records downloaded | 1,088 |
| #8 | #7, filter to peer reviewed | [1,097](https://search-proquest-com.ucsf.idm.oclc.org/recentsearches.recentsearchtabview.recentsearchesgridview.scrolledrecentsearchlist.checkdbssearchlink_0:rerunsearch/44405C7F69B74881PQ/None?site=psycinfo&t:ac=RecentSearches) |
| #7 | #6, date limit 1996-01-01 to 2018-01-09 | 1,206 |
| #6 | 1 AND 2 AND 3 AND 4 AND 5 | 1,250 |
| #5 | ((MJMAINSUBJECT.EXACT("Observation Methods") OR MJMAINSUBJECT.EXACT("Experimental Methods") OR MJMAINSUBJECT.EXACT("Statistical Analysis") OR MJMAINSUBJECT.EXACT("Longitudinal Studies") OR MJMAINSUBJECT.EXACT("Quantitative Methods") OR MJMAINSUBJECT.EXACT("Meta Analysis") OR MJMAINSUBJECT.EXACT("Retrospective Studies") OR MJMAINSUBJECT.EXACT("Prospective Studies") OR MJMAINSUBJECT.EXACT("Time Series") OR MJMAINSUBJECT.EXACT("Risk Factors") OR MJMAINSUBJECT.EXACT("Cohort Analysis") OR MJMAINSUBJECT.EXACT("Statistical Correlation")) OR ab("statistical analysis" OR risk OR controlled OR predictor*OR correlate*OR determinant* OR cofactor* OR "co-factor" OR "risk factor" OR random* OR trial* OR prospective OR retrospective OR intervention OR "case control" OR "cross sectional" OR "cross-sectional" OR observational OR cohort* OR evaluat* OR "non-random" OR "before and after" OR "time series" OR longitudinal* "pre post" OR "pre-post" OR "pre and post" OR "pre/post" OR "time factor" OR "national hiv behavioral surveillance") OR ti("statistical analysis" OR risk OR controlled OR predictor*OR correlate*OR determinant* OR cofactor* OR "co-factor" OR "risk factor" OR random* OR trial* OR prospective OR retrospective OR intervention OR "case control" OR "cross sectional" OR "cross-sectional" OR observational OR cohort* OR evaluat* OR "non-random" OR "before and after" OR "time series" OR longitudinal* "pre post" OR "pre-post" OR "pre and post" OR "pre/post" OR "time factor" OR "national hiv behavioral surveillance")) OR MJMAINSUBJECT.EXACT("Epidemiology") | 1,371,062 |
| #4 | (MJMAINSUBJECT.EXACT("Promiscuity") OR MJMAINSUBJECT.EXACT("Sexual Abstinence") OR MJMAINSUBJECT.EXACT("Same Sex Intercourse") OR MJMAINSUBJECT.EXACT("Sexual Risk Taking") OR MJMAINSUBJECT.EXACT("Safe Sex")) OR MJMAINSUBJECT.EXACT("Condoms") OR ab(serosort* OR "sero-sort*" OR "sero sort*" OR "sexual partner" OR serostatus OR "sero-status" OR "sero status" OR serodiscord* OR "sero-discordant" OR "'sero discordant" OR "HIV discord*" OR seroadapt* OR "sero-adapt*" OR "sero adapt*" OR seroposition* OR "sero-position*" OR "sero position*" OR serocommunicat* OR "sero-communicat*" OR "sero communicat*" OR "partner selection" OR "partner choice" OR "sexual harm reduction" OR "strategic positioning" OR seroguess* OR "sero-guess*" OR "sero guess" OR abstinence OR abstain* OR celiba* OR "safe sex" OR "safer sex" OR "unsafe sex" OR (partner AND ("pre-exposure prophylaxis" OR PrEP OR withdraw* OR "viral load")) OR "risk behav*" OR "risk taking" OR "risk-taking" OR "risk reduction") OR ti(serosort* OR "sero-sort*" OR "sero sort*" OR "sexual partner" OR serostatus OR "sero-status" OR "sero status" OR serodiscord* OR "sero-discordant" OR "'sero discordant" OR "HIV discord*" OR seroadapt* OR "sero-adapt*" OR "sero adapt*" OR seroposition* OR "sero-position*" OR "sero position*" OR serocommunicat* OR "sero-communicat*" OR "sero communicat*" OR "partner selection" OR "partner choice" OR "sexual harm reduction" OR "strategic positioning" OR seroguess* OR "sero-guess*" OR "sero guess" OR abstinence OR abstain* OR celiba* OR "safe sex" OR "safer sex" OR "unsafe sex" OR (partner AND ("pre-exposure prophylaxis" OR PrEP OR withdraw* OR "viral load")) OR "risk behav*" OR "risk taking" OR "risk-taking" OR "risk reduction") OR MJMAINSUBJECT.EXACT("Risk Taking") | 60,728 |
| #3 | (MJMAINSUBJECT.EXACT("Male Homosexuality") OR (MAINSUBJECT.EXACT("Transsexualism") OR MJMAINSUBJECT.EXACT("Transgender")) OR MJMAINSUBJECT.EXACT("Same Sex Intercourse") OR MJMAINSUBJECT.EXACT("Bisexuality")) OR ab(homosex* OR bisex* OR "men who have sex with men*" OR gay OR transgender* OR transsexual* OR transvestite* OR transwomen OR MSM) OR ti(homosex* OR bisex* OR "men who have sex with men*" OR gay OR transgender* OR transsexual* OR transvestite* OR transwomen OR MSM) | 35,324 |
| #2 | MJMAINSUBJECT.EXACT("HIV Testing") OR ab((status OR serostatus) AND know*) OR ti((status OR serostatus) AND know*) OR ab(test result* OR "received result*" OR "received a result" OR "positive result*" OR "test positive" OR "tested positive" OR "HIV counsel*" OR HCT OR VCT OR "HIV positive" OR "HIV-positive" OR "HIV diagnosis") OR ab(test result* OR "received result*" OR "received a result" OR "positive result*" OR "test positive" OR "tested positive" OR "HIV counsel*" OR HCT OR VCT OR "HIV positive" OR "HIV-positive" OR "HIV diagnosis") | 278,114 |
| #1 | MJMAINSUBJECT.EXACT("HIV Testing") OR ab(HIV) OR ab("Human Immunodeficiency") OR ab("Acquired Immun*") AND ti(HIV) OR ti("Human Immunodeficiency") OR ti("Acquired Immun*") | 45,989 |

1. **First Cut Using Title Search in EndNote**

Even when using well-tested database search queries, a certain number of clearly irrelevant results are inevitably returned by any database. These can include records with erroneous subject headings, irrelevant topics that share keywords with our focus, and studies that are clearly out of scope but for which we do not use the database query to exclude results. To address these superfluous records, we employed a strategy we have used successfully in previous reviews: using EndNote’s search feature to identify clearly irrelevant records.

We identified themes and tested keywords that appeared in the titles of clearly irrelevant records in our EndNote library. Concepts and key words listed below include those we have used in previous reviews (such as the “animal” category) and others specific to this review.

One reviewer searched our EndNote library for these title keywords and partitioned results. A second reviewer reviewed the titles of these partitioned studies to ensure that no potentially relevant title has been excluded.

1. **Summary of De-duplication and “first cut” of clearly irrelevant records***

| **Combined results from Embase, PubMed, PsycInfo** | **5,338** |
| --- | --- |
| **Duplicates removed (992 automatically, 304 manually)** | **1,296** |
| Titles: “Animal” records separated | 0 |
| Titles: “In vitro etc.” records separated | 2 |
| Titles: “Drugs” records separated | 81 |
| Titles: “Other conditions” records separated | 445 |
| Titles: “MTCT/obstetrics” records separated | 7 |
| Titles: “Other specific interests” records separated | 71 |
| Titles: “Qualitative” records separated | 59 |
| Titles: “Irrelevant HIV” records separated | 97 |
| Titles: “Trauma” records separated | 53 |
| Titles: “Irrelevant Geographies” records separated | 302 |
| **Total of “clearly irrelevant” records separated** | **1,117** |
| **TOTAL RECORDS SEPARATED in DE-DUPLICATION & FIRST CUT** | **2,413** |
| **RECORDS REMAINING TO BE SCREENED** | **2,925** |

**Details:**

- Combined results from Embase, PubMed, Web of Science: 5,338 total records
- From the total 5,338, I removed 992 duplicates using EndNote’s automated duplicate-finding feature, leaving 4,346.
- From the 4,346, I removed another 304 duplicates based on title + author + year + pagination and/or DOI, leaving 4,042. (Titles of these 304 were not captured in the first attempt due to minor inconsistencies among the databases that precluded EndNote’s automated algorithm from identifying them, such as differing abbreviations for the same journal title.)
- Total duplicate records as of now: 1,296.
- Duplicate records were exported and placed in a separate file.
- Total of additional clearly irrelevant records separated in semi-automated screening by title: 1,117.
- Records remaining to be screened: 2,925.

1. **Title Keyword Searches Within EndNote:**

| **Search** | **Category** (subfolder) | **Records separated** | **Terms used in EndNote, searching only titles**  (asterisk denotes that plural forms & forms with additional subsequent letters were also searched) |
| --- | --- | --- | --- |
| 1 | Animal | 0 | animal* **or** baboon* **or** canine **or** cats **or** chimp* **or** dogs **or** feline **or** guinea pig* **or** hamster* **or** lapine **or** macacque* **or** macaque* **or** mice **or** mouse **or** murine **or** rabbit* **or** rat model **or** rats **or** rhesus **or** simian **or** swine **or** zoonos* |
| 2 | In vitro etc. | 2 | deoxy* **or** in vitro **or** in vivo **or** molecule **or** nano* **or** polymorph* **or** ribosom* |
| 3 | Drugs | 81 | alcohol **or** cocaine **or** crack **or** heroin **or** injecting drug **or** injection drug* **or** intravenous drug* **or** narcotic* **or** needle* **or** opioid* **or** PWID **or** syringe* **or** tobacco |
| 4 | Other conditions | 445 | arthritis **or** cancer* **or** carcinogen* **or** carcinoma* **or** chickenpox **or** dermatolog* **or** digestive **or** ebola **or** Epstein-Barr **or** Epstein Barr **or** eye **or** HCV **or** hematopoie* **or** hepatitis **or** herpes simplex **or** HHV-7 **or** HHV7 **or** HHV-8 **or** HHV8 **or** HPV **or** HSV-2 **or** HSV2 **or** imaging **or** immune reconstitution **or** intestinal **or** Kaposi* **or** Karposi* **or** lymphoma **or** malaria* **or** malignan* **or** necrosis **or** neurolog* **or** neoplasia **or** ocular **or** oncology **or** pap smear  papillomavirus **or** radiolog* **or** skin **or** syphilis **or** transfus* **or** transplant* **or** tuberculosis **or** zika **or** zoster |
| 5 | MTCT/ obstetrics | 7 | antenatal **or** babies **or** breast milk **or** breastmilk **or** caesarean **or** cesarean **or** children **or** fertility **or** fetal **or** foetal **or** infant* **or** infertility **or** intrauterine **or** mother to child **or** mother-to-child **or** MTCT **or** PMTCT **or** miscarriage **or** mothers **or** neonatal **or** neonate* **or** nevirapine **or** perinatal **or** postnatal **or** post-partum **or** postpartum **or** pregnan* **or** prenatal **or** pre-term **or** preterm **or** uterine **or** vertical |
| 6 | Other specific interests | 71 | budget* **or** economic* **or** financial **or** guideline* **or** recommendation* **or** policy **or** mathematical **or** knowledge transfer **or** knowledge translation **or** mass media **or** school-based **or** social market* |
| 7 | Qualitative/ study design | 59 | feasibility **or** qualitative |
| 8 | Irrelevant HIV concepts | 97 | antibod* **or** drug resist* **or** post-exposure or postexposure **or** treatment failure **or** vaccin* |
| 9 | Trauma | 53 | abuse **or** forced sex **or** rape **or** trauma **or** violence |
| 10 | Non-US geographies** | 302 | Angola **or** Botswana **or** Cameroon **or** China **or** Columbia **or** Cote **or** Ecuador **or** El Salvador **or** India **or** Jamaica **or** Kenya **or** Malawi **or** Malaysia **or** Morocco **or** Myanmar **or** Namibia **or** Nigeria **or** Peru **or** Philippines **or** Tanzania **or** Thai* **or** Uganda **or** Vietnam **or** Togo **or** Pakistan  **not** British Columbia **not** USA **not** United States **not** Canada **not** New York |

*Note that because titles of some records may include terms used in ≥1 category, there may be apparent “anomalies” (e.g. a record with “qualitative” and “cocaine” in its title would go to the category for which the internal searches were done first – in this example “drugs” – it would not go to both categories). They are not in fact anomalies.

**This list includes the first 25 clearly irrelevant country names identified in a rapid review of included references. It is not inclusive of all out-of-scope geographies.

##

## Supplemental digital content B: Articles screened at the full text level on changes in sexual practices of men who have sex with men who became aware of HIV diagnosis in the United States

“Sister review searches” were for studies reporting condom outcomes across several risk groups, which we refined to locate sero-adaptive behavior data for MSM only.

**Excluded: Systematic Review Unique from Sister Review (N=1)**

1. Crepaz, N, Marks, G, Liau, A, Mullins, MM, Aupont, LW, Marshall, KJ, Jacobs, ED and Wolitski, RJ. Prevalence of unprotected anal intercourse among HIV-diagnosed MSM in the United States: A meta-analysis. AIDS. 2009; 23(13):1617-1629.

**Excluded: Systematic Review Captured in Sister Review (N=1)**

1. Marks, G, Crepaz, N, Senterfitt, JW and Janssen, RS. Meta-analysis of high-risk sexual behavior in persons aware and unaware they are infected with HIV in the United States: Implications for HIV prevention programs. Journal of Acquired Immune Deficiency Syndromes. 2005; 39(4):446-453.

**Excluded: Primary Studies Captured in Sister Review (N=6)**

1. Steward WT, Remien RH, Higgins JA, Dubrow R, Pinkerton SD, Sikkema KJ, et al. Behavior change following diagnosis with acute/early HIV infection-a move to serosorting with other HIV-infected individuals. the NIMH multisite acute HIV infection study: III. AIDS and Behavior. 2009;13(6):1054-60.
2. Marks G, Millett GA, Bingham T, Bond L, Lauby J, Liau A, et al. Understanding differences in HIV sexual transmission among Latino and Black men who have sex with men: The Brothers y Hermanos Study. AIDS and Behavior. 2009;13(4):682-90.
3. Khosropour CM, Dombrowski JC, Kerani RP, Katz DA, Barbee LA, Golden MR. Changes in Condomless Sex and Serosorting among men who have sex with men after HIV diagnosis. Journal of Acquired Immune Deficiency Syndromes. 2016;73(4):475-81.
4. Gorbach PM, Weiss RE, Jeffries R, Javanbakht M, Drumright LN, Daar ES, et al. Behaviors of recently HIV-infected men who have sex with men in the year postdiagnosis: Effects of drug use and partner types. Journal of Acquired Immune Deficiency Syndromes. 2011;56(2):176-82.
5. Colfax GN, Buchbinder SP, Cornelisse PGA, Vittinghoff E, Mayer K, Celum C. Sexual risk behaviors and implications for secondary HIV transmission during and after HIV seroconversion. AIDS. 2002;16(11):1529-35.
6. CDC. HIV testing and risk behaviors among gay, bisexual, and other men who have sex with men - United States. MMWR Morbidity and mortality weekly report. 2013;62(47):958-62.

**Excluded: during extraction due to lack of outcomes of interest (N=1)**

1. Golden, M. R., et al. (2004). "Importance of sex partner HIV status in HIV risk assessment among men who have sex with men." Journal of Acquired Immune Deficiency Syndromes 36(2): 734-742.

**Excluded: No appropriate comparison group (N=72)**

1. Abler, L, Sikkema, KJ, Watt, MH, Hansen, NB, Wilson, PA and Kochman, A. Depression and HIV Serostatus Disclosure to Sexual Partners among Newly HIV-Diagnosed Men Who Have Sex with Men. AIDS Patient Care and STDs. 2015; 29(10):550-558.
2. Ackers, ML, Greenberg, AE, Lin, CY, Bartholow, BN, Goodman, AH, Longhi, M and Gurwith, M. High and persistent HIV seroincidence in men who have sex with men across 47 U.S. cities. PLoS ONE. 2012; 7(4):
3. Bachmann, LH, Grimley, DM, Chen, H, Aban, I, Hu, J, Zhang, S, Waithaka, YW and Hook, EW, III. Risk behaviours in HIV-positive men who have sex with men participating in an intervention in a primary care setting. International Journal of STD & AIDS. 2009; 20(9):607-612.
4. Bauermeister, JA, Carballo-Diéguez, A, Ventuneac, A and Dolezal, C. Assessing motivations to engage in intentional condomless anal intercourse in HIV risk contexts ("bareback sex") among men who have sex with men. AIDS Education and Prevention. 2009; 21(2):156-168.
5. Beer, L, Oster, AM, Mattson, CL and Skarbinski, J. Disparities in HIV transmission risk among HIV-infected black and white men who have sex with men, United States, 2009. AIDS. 2014; 28(1):105-114.
6. Bingham, T, McFarland, W, Shehan, DA, LaLota, M, Celentano, DD, Koblin, BA, Torian, LV, MacKellar, DA, Valleroy, LA, Secura, GS, Janssen, RS and Roberts, GW. Unrecognized HIV infection, risk behaviors, and perceptions of risk among young Black men who have sex with men--six US cities, 1994-1998. JAMA: Journal of the American Medical Association. 2002; 288(11):1344-1348.
7. Blashill, AJ, O'Cleirigh, C, Mayer, KH, Goshe, BM and Safren, SA. Body mass index, depression and sexual transmission risk behaviors among HIV-positive MSM. AIDS and Behavior. 2012; 16(8):2251-2256.
8. Brown, MJ, Serovich, JM, Kimberly, JA and Umasabor-Bubu, O. Disclosure and Self-Efficacy among HIV-Positive Men Who Have Sex with Men: A Comparison between Older and Younger Adults. AIDS Patient Care and STDs. 2015; 29(11):625-633.
9. Bruce, D, Harper, GW and Suleta, K. Sexual risk behavior and risk reduction beliefs among HIV-positive young men who have sex with men. AIDS and Behavior. 2013; 17(4):1515-1523.
10. Calzavara, L, Burchell, AN, Remis, RS, Major, C, Corey, P, Myers, T, Millson, M, Wallace, E, Ostrowski, M, McGee, F, Read, S, Rachlis, A, Haubrich, D, Logue, K, Trow, R, Tharao, E, Crossman, C, Gough, K and Sandstrom, P. Delayed application of condoms is a risk factor for human immunodeficiency virus infection among homosexual and bisexual men. American Journal of Epidemiology. 2003; 157(3):210-217.
11. Carrico, AW, Chesney, MA, Johnson, MO, Morin, SF, Neilands, TB, Remien, RH, Rotheram-Borus, MJ and Wong, FL. Randomized controlled trial of a cognitive-behavioral intervention for HIV-positive persons: An investigation of treatment effects on psychosocial adjustment. AIDS and Behavior. 2009; 13(3):555-563.
12. CDC. HIV infection among young black men who have sex with men--Jackson, Mississippi, 2006-2008. MMWR. Morbidity and mortality weekly report. 2009; 58(4):77-81.
13. CDC. HIV prevalence, unrecognized infection, and HIV testing among men who have sex with men--five U.S. cities, June 2004-April 2005. MMWR Morb Mortal Wkly Rep. 2005; 54(24):597-601.
14. CDC. HIV testing among men who have sex with men--21 cities, United States, 2008. MMWR Morb Mortal Wkly Rep. 2011; 60(21):694-9.
15. Clatts, MC, Rodríguez-Díaz, CE, García, H, Vargas-Molina, RL, Jovet-Toledo, GG and Goldsamt, L. A preliminary profile of HIV risk in a clinic-based sample of MSM in Puerto Rico: Implications for sexual health promotion interventions. Puerto Rico Health Sciences Journal. 2012; 31(3):154-160.
16. Conroy, AA, Gamarel, KE, Neilands, TB, Sauceda, JA, Darbes, LA, Dilworth, SE, Taylor, JM and Johnson, MO. Partner reports of HIV viral suppression predict sexual behavior in serodiscordant male couples. JAIDS Journal of Acquired Immune Deficiency Syndromes. 2016; 73(2):e31-e33.
17. Crepaz, N and Marks, G. Serostatus disclosure, sexual communication and safer sex in HIV-positive men. AIDS Care - Psychological and Socio-Medical Aspects of AIDS/HIV. 2003; 15(3):379-387.
18. Crepaz, N, Marks, G, Mansergh, G, Murphy, S, Miller, LC and Appleby, PR. Age-related risk for HIV infection in men who have sex with men: Examination of behavioral, relationship, and serostatus variables. AIDS Education and Prevention. 2000; 12(5):405-415.
19. Crosby, R, Salazar, LF and Mettey, A. Gay men who attend sex resorts: A typology associated with high-risk sexual behaviour. International Journal of STD and AIDS. 2005; 16(2):158-162.
20. Davey, DJ, Beymer, M, Roberts, CP, Bolan, RK and Klausner, JD. Differences in risk behavior and demographic factors between men who have sex with men with acute and nonacute human immunodeficiency virus infection in a community-based testing program in los angeles. Journal of Acquired Immune Deficiency Syndromes. 2017; 74(4):e97-e103.
21. Denning, PH and Campsmith, ML. Unprotected Anal Intercourse Among HIV-Positive Men Who Have a Steady Male Sex Partner With Negative or Unknown HIV Serostatus. American Journal of Public Health. 2005; 95(1):152-158.
22. Dombrowski, JC, Harrington, RD and Golden, MR. Evidence for the long-term stability of HIV transmission-associated sexual behavior after HIV diagnosis. Sexually Transmitted Diseases. 2013; 40(1):41-45.
23. Dorell, CG, Sutton, MY, Oster, AM, Hardnett, F, Thomas, PE, Gaul, ZJ, Mena, LA and Heffelfinger, JD. Missed opportunities for HIV testing in health care settings among young African American men who have sex with men: Implications for the HIV epidemic. AIDS Patient Care and STDs. 2011; 25(11):657-664.
24. Drabkin, AS, Sikkema, KJ, Wilson, PA, Meade, CS, Hansen, NB, Delorenzo, A, Kochman, A, MacFarlane, JC, Watt, MH, Aunon, FM, Ranby, KW and Mayer, G. Risk patterns preceding diagnosis among newly HIV-diagnosed men who have sex with men in New York City. AIDS Patient Care and STDs. 2013; 27(6):333-341.
25. Eaton, LA, Cherry, C, Cain, D and Pope, H. A novel approach to prevention for at-risk HIV-negative men who have sex with men: creating a teachable moment to promote informed sexual decision-making. American journal of public health. 2011; 101(3):539-545.
26. Eaton, LA, Kalichman, SC, O'Connell, DA and Karchner, WD. A strategy for selecting sexual partners believed to pose little/no risks for HIV: Serosorting and its implications for HIV transmission. AIDS Care - Psychological and Socio-Medical Aspects of AIDS/HIV. 2009; 21(10):1279-1288.
27. Eaton, LA, Maksut, JL, Gamarel, KE, Siembida, EJ, Driffin, DD and Baldwin, R. Online Sex Partner Meeting Venues as a Risk Factor for Testing HIV Positive among a Community-Based Sample of Black Men Who Have Sex with Men. Sexually Transmitted Diseases. 2016; 43(6):360-364.
28. Eaton, LA, West, TV, Kenny, DA and Kalichman, SC. HIV transmission risk among HIV seroconcordant and serodiscordant couples: Dyadic processes of partner selection. AIDS and Behavior. 2009; 13(2):185-195.
29. Ellen, JM, Greenberg, L, Willard, N, Stines, S, Korelitz, J and Boyer, CB. Cross-sectional survey comparing HIV risk behaviours of adolescent and young adult men who have sex with men only and men who have sex with men and women in the US and Puerto Rico. Sexually Transmitted Infections. 2015; 91(6):458-461.
30. Emlet, CA, Shiu, C, Kim, HJ and Fredriksen-Goldsen, K. Bouncing Back: Resilience and Mastery Among HIV-Positive Older Gay and Bisexual Men. Gerontologist. 2017; 57(suppl 1):S40-s49.
31. Facente, SN, Pilcher, CD, Hartogensis, WE, Klausner, JD, Philip, SS, Louie, B, Christopoulos, KA, Dowling, T and Colfax, GN. Performance of risk-based criteria for targeting acute hiv screening in san francisco. PLoS ONE. 2011; 6(7):
32. Fendrich, M, Mackesy-Amiti, ME, Johnson, TP and Pollack, LM. Sexual risk behavior and drug use in two Chicago samples of men who have sex with men: 1997 vs. 2002. J Urban Health. 2010; 87(3):452-66.
33. Fernández, MI, Perrino, T, Bowen, GS, Royal, S and Varga, L. Repeat HIV testing among Hispanic men who have sex with men - A sign of risk, prevention, or reassurance? AIDS Education and Prevention. 2003; 15(SUPPL. 15):105-116.
34. Finlayson, TJ, Le, B, Smith, A, Bowles, K, Cribbin, M, Miles, I, Oster, AM, Martin, T, Edwards, A and Dinenno, E. HIV risk, prevention, and testing behaviors among men who have sex with men--National HIV Behavioral Surveillance System, 21 U.S. cities, United States, 2008. MMWR Surveill Summ. 2011; 60(14):1-34.
35. Fisher, HH, Purcell, DW, Hoff, CC, Parsons, JT and O'Leary, A. Recruitment source and behavioral risk patterns of HIV-positive men who have sex with men. AIDS and Behavior. 2006; 10(5):553-561.
36. Fisher, JD, Willcutts, DLK, Misovich, SJ and Weinstein, B. Dynamics of sexual risk behavior in HIV-infected men who have sex with men. AIDS and Behavior. 1998; 2(2):101-113.
37. Fisher, MP, Ramchand, R, Bana, S and Iguchi, MY. Risk behaviors among HIV-positive gay and bisexual men at party-oriented vacations. Journal of Studies on Alcohol and Drugs. 2013; 74(1):158-167.
38. Forney, JC and Miller, RL. Risk and protective factors related to HIV-risk behavior: A comparison between HIV-positive and HIV-negative young men who have sex with men. AIDS Care. 2012; 24(5):544-552.
39. Friedman, MR, Kurtz, SP, Buttram, ME, Wei, C, Silvestre, AJ and Stall, R. HIV risk among substance-using men who have sex with men and women (MSMW): findings from South Florida. AIDS and behavior. 2014; 18(1):111-119.
40. Fuqua, V, Chen, Y-H, Packer, T, Dowling, T, Ick, TO, Nguyen, B, Colfax, GN and Raymond, HF. Using social networks to reach Black MSM for HIV testing and linkage to care. AIDS and Behavior. 2012; 16(2):256-265.
41. German, D, Brady, K, Kuo, I, Opoku, J, Flynn, C, Patrick, R, Park, JN, Adams, J, Carroll, M, Simmons, R, Smith, CR and Davis, WW. Characteristics of Black Men Who Have Sex With Men in Baltimore, Philadelphia, and Washington, D.C.: Geographic Diversity in Socio-Demographics and HIV Transmission Risk. Journal of acquired immune deficiency syndromes (1999). 2017; 75(S296-S308.
42. Glick, SN and Golden, MR. Early male partnership patterns, social support, and sexual risk behavior among young men who have sex with men. AIDS and behavior. 2014; 18(8):1466-1475.
43. Glynn, TR, Operario, D, Montgomery, M, Almonte, A and Chan, PA. The Duality of Oral Sex for Men Who Have Sex with Men: An Examination into the Increase of Sexually Transmitted Infections Amid the Age of HIV Prevention. AIDS Patient Care and STDs. 2017; 31(6):261-267.
44. Golden, MR, Wood, RW, Buskin, SE, Fleming, M and Harrington, RD. Ongoing risk behavior among persons with HIV in medical care. AIDS and Behavior. 2007; 11(5):726-735.
45. Gorbach, PM, Drumright, LN, Daar, ES and Little, SJ. Transmission behaviors of recently HIV-infected men who have sex with men. J Acquir Immune Defic Syndr. 2006; 42(1):80-5.
46. Green, N, Hoenigl, M, Morris, S and Little, SJ. Risk behavior and sexually transmitted infections among transgender women and men undergoing community-based screening for acute and early HIV infection in San Diego. Medicine (United States). 2015; 94(41):
47. Grey, JA, Rothenberg, R, Sullivan, PS and Rosenberg, ES. Racial differences in the accuracy of perceived partner HIV status among men who have sex with men (MSM) in Atlanta, Georgia. Journal of the International Association of Providers of AIDS Care. 2015; 14(1):26-32.
48. Grov, C, DeBusk, JA, Bimbi, DS, Golub, SA, Nanin, JE and Parsons, JT. Barebacking, the Internet, and harm reduction: An intercept survey with gay and bisexual men in Los Angeles and New York City. AIDS and Behavior. 2007; 11(4):527-536.
49. Grov, C, Rendina, HJ and Parsons, JT. Comparing three cohorts of MSM sampled via sex parties, bars/clubs, and Craigslist.org: Implications for researchers and providers. AIDS Education and Prevention. 2014; 26(4):362-382.
50. Grov, C, Rendina, HJ, Moody, RL, Ventuneac, A and Parsons, JT. HIV Serosorting, Status Disclosure, and Strategic Positioning among Highly Sexually Active Gay and Bisexual Men. AIDS Patient Care and STDs. 2015; 29(10):559-568.
51. Grov, C, Rendina, HJ, Ventuneac, A and Parsons, JT. HIV risk in group sexual encounters: An event‐level analysis from a national online survey of MSM in the U. S. Journal of Sexual Medicine. 2013; 10(9):2285-2294.
52. Haas, SM, Perazzo, JD, Ruffino, AH, Ancona, RM and Lyons, M. The know*now project: Facilitated serosorting in HIV-status sexual partner communication. AIDS Education and Prevention. 2017; 29(5):432-442.
53. Hakre, S, Scoville, SL, Pacha, LA, Peel, SA, Kim, JH, Michael, NL, Cersovsky, SB and Scott, PT. Sexual risk behaviors of HIV seroconverters in the US army, 2012-2014. Journal of Acquired Immune Deficiency Syndromes. 2015; 70(4):456-461.
54. Halkitis, PN, Green, KA and Carragher, DJ. Methamphetamine Use, Sexual Behavior, and HIV Seroconversion. Journal of Gay & Lesbian Psychotherapy. 2006; 10(3-4):95-109.
55. Hart, TA, James, CA, Hagan, CMP and Boucher, E. HIV optimism and high-risk sexual behavior in two cohorts of men who have sex with men. JANAC: Journal of the Association of Nurses in AIDS Care. 2010; 21(5):439-443.
56. Hart, TA, James, CA, Purcell, DW and Farber, E. Social anxiety and HIV transmission risk among HIV-seropositive male patients. AIDS Patient Care and STDs. 2008; 22(11):879-886.
57. Hart, TA, Stratto, N, Coleman, TA, Wilson, HA, Simpson, SH, Julien, RE, Hoe, D, Leahy, B, Maxwell, J and Adam, BD. A pilot trial of a sexual health counseling intervention for HIV-positive gay and bisexual men who report anal sex without condoms. PLoS ONE. 2016; 11(4):
58. Hoff, CC, Chakravarty, D, Beougher, SC, Neilands, TB and Darbes, LA. Relationship characteristics associated with sexual risk behavior among MSM in committed relationships. AIDS Patient Care and STDs. 2012; 26(12):738-745.
59. Horvath, KJ, Oakes, JM and Rosser, BR. Sexual negotiation and HIV serodisclosure among men who have sex with men with their online and offline partners. Journal of urban health : bulletin of the New York Academy of Medicine. 2008; 85(5):744-758.
60. Huebner, DM, Binson, D, Woods, WJ, Dilworth, SE, Neilands, TB and Grinstead, O. Bathhouse-based voluntary counseling and testing is feasible and shows preliminary evidence of effectiveness. Journal of Acquired Immune Deficiency Syndromes. 2006; 43(2):239-246.
61. Khosropour, CM, Dombrowski, JC, Swanson, F, Kerani, RP, Katz, DA, Barbee, LA, Hughes, JP, Manhart, LE and Golden, MR. Trends in serosorting and the association with HIV/STI risk over time among men who have sex with men. Journal of Acquired Immune Deficiency Syndromes. 2016; 72(2):189-197.
62. Koblin, BA, Torian, LV, Guilin, V, Ren, L, MacKellar, DA and Valleroy, LA. High prevalence of HIV infection among young men who have sex with men in New York City. Aids. 2000; 14(12):1793-800.
63. Landovitz, RJ, Tran, TTT, Cohn, SE, Ofotokun, I, Godfrey, C, Kuritzkes, DR, Lennox, JL, Currier, JS and Ribaudo, HJ. HIV transmission risk behavior in a cohort of HIV-infected treatment-naïve men and women in the United States. AIDS and Behavior. 2016; 20(12):2983-2995.
64. Lauby, JL, Millett, GA, LaPollo, AB, Bond, L, Murrill, CS and Marks, G. Sexual risk behaviors of HIV-positive, HIV-negative, and serostatus-unknown Black men who have sex with men and women. Archives of Sexual Behavior. 2008; 37(5):708-719.
65. MacKellar, DA, Valleroy, LA, Secura, GM, Behel, S, Bingham, T, Celentano, DD, Koblin, BA, Lalota, M, McFarland, W, Shehan, D, Thiede, H, Torian, LV and Janssen, RS. Unrecognized HIV infection, risk behaviors, and perceptions of risk among young men who have sex with men: opportunities for advancing HIV prevention in the third decade of HIV/AIDS. J Acquir Immune Defic Syndr. 2005; 38(5):603-14.
66. Margolis, AD, Joseph, H, Hirshfield, S, Chiasson, MA, Belcher, L and Purcell, DW. Anal intercourse without condoms among HIV-positive men who have sex with men recruited from a sexual networking web site, United States. Sexually Transmitted Diseases. 2014; 41(12):749-755.
67. Marks, G and Crepaz, N. HIV-positive men's sexual practices in the context of self-disclosure of HIV status. Journal of Acquired Immune Deficiency Syndromes. 2001; 27(1):79-85.
68. Maung Maung, T, Chen, B, Moore, DM, Chan, K, Kanters, S, Michelow, W, Hogg, RS, Nakamura, N, Robert, W, Gustafson, R, Gilbert, M and ManCount Study, T. Risks for HIV and other sexually transmitted infections among Asian men who have sex with men in Vancouver, British Columbia: a cross-sectional survey. BMC public health. 2013; 13(763.
69. Pollack, LM, Woods, WJ, Blair, J and Binson, D. Presence of an HIV testing program lowers the prevalence of unprotected insertive anal intercourse inside a gay bathhouse among HIV-negative and HIV-unknown patrons. Journal of HIV/AIDS & Social Services. 2014; 13(3):306-323.
70. Posner, SF and Marks, G. Prevalence of high-risk sex among HIV-positive gay and bisexual men: A longitudinal analysis. American Journal of Preventive Medicine. 1996; 12(6):472-477.
71. Sikkema, KJ, Abler, L, Hansen, NB, Wilson, PA, Drabkin, AS, Kochman, A, MacFarlane, JC, DeLorenzo, A, Mayer, G, Watt, MH and Nazareth, W. Positive choices: outcomes of a brief risk reduction intervention for newly HIV-diagnosed men who have sex with men. AIDS Behav. 2014; 18(9):1808-19.
72. Washington, TA, Robles, G and Malotte, K. Factors associated with HIV-testing history among Black men who have sex with men (BMSM) in Los Angeles County. Behav Med. 2013; 39(3):52-9.

**Excluded: Document type (N=6)**

1. Fisher, HH, Purcell, DW, Hoff, CC, Parsons, JT and O'Leary, A. "Recruitment source and behavioral risk patterns of HIV-positive men who have sex with men": Erratum. AIDS and Behavior. 2007; 11(2):335.
2. Golden, MR, Stekler, J, Hughes, JP and Wood, RW. "HIV serosorting in men who have sex with men: Is it safe?": Erratum. JAIDS Journal of Acquired Immune Deficiency Syndromes. 2008; 49(4):464.
3. Herbst, JH, Jacobs, ED, Finlayson, TJ, McKleroy, VS, Neumann, MS and Crepaz, N. Estimating HIV prevalence and risk behaviors of transgender persons in the United States: a systematic review. AIDS Behav. 2008; 12(1):1-17.
4. Lewis, NM and Wilson, K. HIV risk behaviours among immigrant and ethnic minority gay and bisexual men in North America and Europe: A systematic review. Social Science and Medicine. 2017; 179(115-128.
5. Phillips, G, II, Wohl, A, Xavier, J, Jones, K and Hidalgo, J. Epidemiologic data on young men of color who have sex with men. AIDS Patient Care and STDs. 2011; 25(Sup1):S3-S8.
6. van Kesteren, NMC, Hospers, HJ and Kok, G. Sexual risk behavior among HIV-positive men who have sex with men: A literature review. Patient Education and Counseling. 2007; 65(1):5-20.

**Excluded: No HIV-positive participants in analysis (N=5)**

1. Alexovitz, KA, Merchant, RC, Clark, MA, Liu, T, Rosenberger, JG, Bauermeister, J and Mayer, KH. Discordance of voluntary HIV testing with HIV sexual risk-taking and self-perceived HIV infection risk among social media-using black, Hispanic, and white young-men-who-have-sex-with-men (YMSM). AIDS Care - Psychological and Socio-Medical Aspects of AIDS/HIV. 2018; 30(1):81-85.
2. Dilley, JW, Schwarcz, S, Murphy, J, Joseph, C, Vittinghoff, E and Scheer, S. Efficacy of personalized cognitive counseling in men of color who have sex with men: secondary data analysis from a controlled intervention trial. AIDS and behavior. 2011; 15(5):970-975.
3. Dilley, JW, Woods, WJ, Loeb, L, Nelson, K, Sheon, N, Mullan, J, Adler, B, Chen, S and McFarland, W. Brief cognitive counseling with HIV testing to reduce sexual risk among men who have sex with men: Results from a randomized controlled trial using paraprofessional counselors. Journal of Acquired Immune Deficiency Syndromes. 2007; 44(5):569-577.
4. Dilley, JW, Woods, WJ, Sabatino, J, Lihatsh, T, Adler, B, Casey, S, Rinaldi, J, Brand, R and McFarland, W. Changing sexual behavior among gay male repeat testers for HIV: A randomized, controlled trial of a single-session intervention. Journal of Acquired Immune Deficiency Syndromes. 2002; 30(2):177-186.
5. Eaton, LA, Kalichman, SC, Cain, DN, Cherry, C, Stearns, HL, Amaral, CM, Flanagan, JA and Pope, HL. Serosorting Sexual Partners and Risk for HIV Among Men Who Have Sex with Men. American Journal of Preventive Medicine. 2007; 33(6):479-485.

**Excluded: No outcomes of interest (N=10)**

1. Blair, JM, Fagan, JL, Frazier, EL, Do, A, Bradley, H, Valverde, EE, McNaghten, A, Beer, L, Zhang, S, Huang, P, Mattson, CL, Freedman, MS, Johnson, CH, Sanders, CC, Spruit-McGoff, KE, Heffelfinger, JD and Skarbinski, J. Behavioral and clinical characteristics of persons receiving medical care for HIV infection - Medical Monitoring Project, United States, 2009. Morbidity and mortality weekly report. Surveillance summaries (Washington, D.C. : 2002). 2014; 63 Suppl 5(1-22).
2. CDC. High-Risk Sexual Behavior by HIV-Positive Men Who Have Sex With Men--16 Sites, United States, 2000-2002. JAMA: Journal of the American Medical Association. 2004; 292(19):2333-2334.
3. Crosby, RA, Mena, L and Geter, A. Are HIV-positive young black MSM having safer sex than those who are HIV-negative? International Journal of STD and AIDS. 2017; 28(5):441-446.
4. Kelly, JA, St Lawrence, JS, Tarima, SS, DiFranceisco, WJ and Amirkhanian, YA. Correlates of Sexual HIV Risk Among African American Men Who Have Sex With Men. American journal of public health. 2016; 106(1):96-102.
5. Myers, T, Godin, G, Lambert, J, Calzavara, L and Locker, D. Sexual risk and HIV-testing behaviour by gay and bisexual men in Canada. AIDS Care. 1996; 8(3):297-309.
6. Ng, BE, Moore, D, Michelow, W, Hogg, R, Gustafson, R, Robert, W, Kanters, S, Thumath, M, McGuire, M and Gilbert, M. Relationship between disclosure of same-sex sexual activity to providers, HIV diagnosis and sexual health services for men who have sex with men in Vancouver, Canada. Can J Public Health. 2014; 105(3):e186-91.
7. Reisen, CA, Zea, MC, Bianchi, FT and Poppen, PJ. Characteristics of Latino MSM who have sex in public settings. AIDS Care. 2011; 23(4):456-459.
8. Rendina, HJ, Jimenez, RH, Grov, C, Ventuneac, A and Parsons, JT. Patterns of lifetime and recent HIV testing among men who have sex with men in New York City who use Grindr. AIDS Behav. 2014; 18(1):41-9.
9. Sumartojo, E, Lyles, C, Choi, K, Clark, L, Collins, C, Grey, CG, Lin, LS, Peterson, JL and Remafedi, G. Prevalence and correlates of HIV testing in a multi-site sample of young men who have sex with men. AIDS Care. 2008; 20(1):1-14.
10. Tieu, HV, Nandi, V, Frye, V, Stewart, K, Oquendo, H, Bush, B, Cerda, M, Hoover, DR, Ompad, D and Koblin, BA. Concurrent partnerships and HIV risk among men who have sex with men in New York City. Sexually Transmitted Diseases. 2014; 41(3):200-208.

**Excluded: Not from US (N=4)**

1. Davis, M, Hart, G, Bolding, G, Sherr, L and Elford, J. Sex and the internet: Gay men, risk reduction and serostatus. Culture, Health and Sexuality. 2006; 8(2):161-174.
2. Leaity, S, Sherr, L, Wells, H, Evans, A, Miller, R, Johnson, M and Elford, J. Repeat HIV testing: High-risk behaviour or risk reduction strategy? AIDS. 2000; 14(5):547-552.
3. Moore DM, Kanters S, Michelow W, Gustafson R, Hogg RS, Kwag M, et al. Implications for HIV prevention programs from a serobehavioural survey of men who have sex with men in Vancouver, British Columbia: the ManCount study. Canadian journal of public health Revue canadienne de santé publique. 2012;103(2):142-6.
4. Suzan-Monti, M, Lorente, N, Demoulin, B, Marcellin, F, Preau, M, Dray-Spira, R, Lert, F and Spire, B. Sexual risk behaviour among people living with HIV according to the biomedical risk of transmission: results from the ANRS-VESPA2 survey. J Int AIDS Soc. 2016; 19(1):20095.

**Exclude: Control condition HIV-negative (N=21)**

1. Hays, RB, Kegeles, SM and Coates, TJ. Unprotected sex and HIV risk taking among young gay men within boyfriend relationships. AIDS Education and Prevention. 1997; 9(4):314-329.
2. Hays, RB, Paul, J, Ekstrand, M, Kegeles, SM, Stall, R and Coates, TJ. Actual versus perceived HIV status, sexual behaviors and predictors of unprotected sex among young gay and bisexual men who identify as HIV-negative, HIV-positive and untested. AIDS. 1997; 11(12):1495-1502.
3. Hurt, CB, Matthews, DD, Calabria, MS, Green, KA, Adimora, AA, Golin, CE and Hightow-Weidman, LB. Sex with older partners is associated with primary HIV infection among men who have sex with men in North Carolina. J Acquir Immune Defic Syndr. 2010; 54(2):185-90.
4. Joseph, HA, Pan, Y, Mendoza, M, Harawa, NT, Lauby, J, Hosek, SG, Bluthenthal, RN, Milnamow, M, Fernandez, MI, Jeffries, WL, Belcher, L and Millett, GA. Hiv acquisition and transmission potential among african american men who have sex with men and women in three u.S. Cities. Archives of Sexual Behavior. 2017;
5. Kalichman, SC, Eaton, L, Cain, D, Cherry, C, Fuhrel, A, Kaufman, M and Pope, H. Changes in HIV Treatment Beliefs and Sexual Risk Behaviors Among Gay and Bisexual Men, 1997-2005. Health Psychology. 2007; 26(5):650-656.
6. Kalichman, SC, Eaton, L, White, D, Cherry, C, Pope, H, Cain, D and Kalichman, MO. Beliefs about treatments for HIV/AIDS and sexual risk behaviors among men who have sex with men, 1997-2006. Journal of Behavioral Medicine. 2007; 30(6):497-503.
7. Kelly, JA, St. Lawrence, JS, Amirkhanian, YA, DiFranceisco, WJ, Anderson-Lamb, M, Garcia, LI and Nguyen, MT. Levels and predictors of HIV risk behavior among Black men who have sex with men. AIDS Education and Prevention. 2013; 25(1):49-61.
8. Lauby, JL, Marks, G, Bingham, T, Liu, K-L, Liau, A, Stueve, A and Millett, GA. Having supportive social relationships is associated with reduced risk of unrecognized HIV infection among Black and Latino men who have sex with men. AIDS and Behavior. 2012; 16(3):508-515.
9. Mimiaga, MJ, Reisner, SL, Cranston, K, Isenberg, D, Bright, D, Daffin, G, Bland, S, Driscoll, MA, Vanderwarker, R, Vega, B and Mayer, KH. Sexual mixing patterns and partner characteristics of black msm in massachusetts at increased risk for HIV infection and transmission. Journal of Urban Health. 2009; 86(4):602-623.
10. Ostrow, DE, Fox, KJ, Chmiel, JS, Silvestre, A, Visscher, BR, Vanable, PA, Jacobson, LP and Strathdee, SA. Attitudes towards highly active antiretroviral therapy are associated with sexual risk taking among HIV-infected and uninfected homosexual men. AIDS. 2002; 16(5):775-780.
11. Paz-Bailey, G, Mendoza, MCB, Finlayson, T, Wejnert, C, Le, B, Rose, C, Raymond, HF, Prejean, J, Taussig, J, Gern, R, Hoyte, T, Salazar, L, White, J, Todd, J, Bautista, G, Flynn, C, Sifakis, F, German, D, Isenberg, D, Driscoll, M, Hurwitz, E, Miminos, M, Doherty, R, Wittke, C, Prachand, N, Benbow, N, Melville, S, Pannala, P, Yeager, R, Sayegh, A, Dyer, J, Sheu, S, Novoa, A, Thrun, M, Al-Tayyib, A, Wilmoth, R, Higgins, E, Griffin, V, Mokotoff, E, Mac-Master, K, Wolverton, M, Risser, J, Rehman, H, Padgett, P, Bingham, T, Sey, EK, LaLota, M, Metsch, L, Forrest, D, Beck, D, Cardenas, G, Nemeth, C, Anderson, BJ, Watson, CA, Smith, L, Orleans, N, Robinson, WT, Gruber, D, Barak, N, Murrill, C, Neaigus, A, Jenness, S, Hagan, H, Reilly, KH, Wendel, T, Cross, H, Bolden, B, D'Errico, S, Wogayehu, A, Godette, H, Brady, KA, Kirkland, A, Sifferman, A, Miguelino-Keasling, V, Velasco, A, Tovar, V, Raymond, HF, De León, SM, Rolón-Colón, Y, Marzan, M, Courogen, M, Jaenicke, T, Thiede, H, Burt, R, Jia, Y, Opoku, J, Sansone, M, West, T, Magnus, M and Kuo, I. Trends in condom use among MSM in the United States: The role of antiretroviral therapy and seroadaptive strategies. AIDS. 2016; 30(12):1985-1990.
12. Robinson, BBE, Galbraith, JS, Swinburne Romine, RE, Zhang, Q and Herbst, JH. Differences between HIV-positive and HIV-negative African American men who have sex with men in two major U.S. metropolitan areas. Archives of Sexual Behavior. 2013; 42(2):267-278.
13. Scheer, S, Kellogg, T, Klausner, JD, Schwarcz, S, Colfax, G, Bernstein, K, Louie, B, Dilley, JW, Hecht, J, Truong, HHM, Katz, MH and McFarland, W. HIV is hyperendemic among men who have sex with men in San Francisco: 10-year trends in HIV incidence, HIV prevalence, sexually transmitted infections and sexual risk behaviour. Sexually Transmitted Infections. 2008; 84(6):493-498.
14. Snowden, JM, Wei, C, McFarland, W and Raymond, HF. Prevalence, correlates and trends in seroadaptive behaviours among men who have sex with men from serial cross-sectional surveillance in San Francisco, 2004-2011. Sex Transm Infect. 2014; 90(6):498-504.
15. Starks, TJ, Gamarel, KE and Johnson, MO. V serodiscordant relationships. Archives of Sexual Behavior. 2014; 43(1):139-147.
16. Taylor, BS, Chiasson, MA, Scheinmann, R, Hirshfield, S, Humberstone, M, Remien, RH, Wolitski, RJ and Wong, T. Results from two online surveys comparing sexual risk behaviors in Hispanic, black, and white men who have sex with men. AIDS Behav. 2012; 16(3):644-52.
17. Thiede, H, Jenkins, RA, Carey, JW, Hutcheson, R, Thomas, KK, Stall, RD, White, E, Allen, I, Mejia, R and Golden, MR. Determinants of recent HIV infection among Seattle-area men who have sex with men. American journal of public health. 2009; 99 Suppl 1(S157-164.
18. Tieu, HV, Murrill, C, Xu, G and Koblin, BA. Sexual Partnering and HIV Risk among Black Men Who Have Sex with Men: New York City. Journal of Urban Health. 2009; 1-9.
19. Truong, H-HM, Fatch, R, Raymond, HF and McFarland, W. HIV treatment and re-infection beliefs predict sexual risk behavior of men who have sex with men. AIDS Education and Prevention. 2017; 29(3):218-227.
20. Truong, HM, Fatch, R, Raymond, HF and McFarland, W. HIV Treatment and Re-infection Beliefs Predict Sexual Risk Behavior of Men Who Have Sex With Men. AIDS Educ Prev. 2017; 29(3):218-227.
21. Xia, Q, Molitor, F, Osmond, DH, Tholandi, M, Pollack, LM, Ruiz, JD and Catania, JA. Knowledge of sexual partner's HIV serostatus and serosorting practices in a California population-based sample of men who have sex with men. AIDS. 2006; 20(16):2081-2089.

**No reporting of outcomes by serostatus (N=13)**

1. Hoenigl, M, Anderson, CM, Green, N, Mehta, SR, Smith, DM and Little, SJ. Repeat HIV-testing is associated with an increase in behavioral risk among men who have sex with men: a cohort study. BMC Med. 2015; 13(218.
2. Joseph, HA, Flores, SA, Parsons, JT and Purcell, DW. Beliefs about transmission risk and vulnerability, treatment adherence, and sexual risk behavior among a sample of HIV-positive men who have sex with men. AIDS Care. 2010; 22(1):29-39.
3. Koblin, BA, Mayer, KH, Eshleman, SH, Wang, L, Mannheimer, S, del Rio, C, Shoptaw, S, Magnus, M, Buchbinder, S, Wilton, L, Liu, TY, Cummings, V, Piwowar-Manning, E, Fields, SD, Griffith, S, Elharrar, V and Wheeler, D. Correlates of HIV acquisition in a cohort of Black men who have sex with men in the United States: HIV prevention trials network (HPTN) 061. PLoS One. 2013; 8(7):e70413.
4. Kuruc, JD, Cope, AB, Sampson, LA, Gay, CL, Ashby, RM, Foust, EM, Brinson, M, Barnhart, JE, Margolis, D, Miller, WC, Leone, PA and Eron, JJ. Ten Years of Screening and Testing for Acute HIV Infection in North Carolina. J Acquir Immune Defic Syndr. 2016; 71(1):111-9.
5. McCree, DH, Johnson, W, Baytop, C and Royal, S. Risk behaviors and testing history of African American MSM: Implications for prevention. Journal of the National Medical Association. 2016; 108(4):220-224.
6. Nelson, KM, Thiede, H, Hawes, SE, Golden, MR, Hutcheson, R, Carey, JW, Kurth, A and Jenkins, RA. Why the Wait? Delayed HIV Diagnosis among Men Who Have Sex with Men. Journal of Urban Health. 2010; 1-14.
7. Oster, AM, Wiegand, RE, Sionean, C, Miles, IJ, Thomas, PE, Melendez-Morales, L, Le, BC and Millett, GA. Understanding disparities in HIV infection between black and white MSM in the United States. Aids. 2011; 25(8):1103-12.
8. Pathela, P, Jamison, K, Braunstein, SL, Schillinger, JA, Varma, JK and Blank, S. Incidence and predictors of HIV infection among men who have sex with men attending public sexually transmitted disease clinics, New York City, 2007–2012. AIDS and Behavior. 2017; 21(5):1444-1451.
9. Przybyla, S, Golin, C, Widman, L, Grodensky, C, Earp, JA and Suchindran, C. Examining the role of serostatus disclosure on unprotected sex among people living with HIV. AIDS Patient Care and STDs. 2014; 28(12):677-684.
10. Ruiz, J, Facer, M and Sun, RK. Risk factors for human immunodeficiency virus infection and unprotected anal intercourse among young men who have sex with men. Sexually Transmitted Diseases. 1998; 25(2):100-107.
11. Strathdee, SA, Martindale, SL, Cornelisse, PGA, Miller, ML, Craib, KJP, Schechter, MT, O'Shaughnessy, MV and Hogg, RS. HIV infection and risk behaviours among young gay and bisexual men in Vancouver. Canadian Medical Association Journal. 2000; 162(1):21-25.
12. Wilson, PA, Kahana, SY, Fernandez, MI, Harper, GW, Mayer, K, Wilson, CM and Hightow-Weidman, LB. Sexual risk behavior among virologically detectable human immunodeficiency virus-infected young men who have sex with men. JAMA Pediatrics. 2016; 170(2):125-131.
13. Wu, H, Hightow-Weidman, LB, Gay, CL, Zhang, X, Beagle, S, Hall, L, Jackson, T, Marmorino, J, Do, AN and Peters, PJ. Unreported Male Sex Partners Among Men with Newly Diagnosed HIV Infection - North Carolina, 2011-2013. MMWR Morb Mortal Wkly Rep. 2015; 64(37):1037-41.

**Insufficient quantitative analysis (N=1)**

1. Balán, IC, Carballo-Diéguez, A, Frasca, T, Dolezal, C and Ibitoye, M. The impact of rapid HIV home test use with sexual partners on subsequent sexual behavior among men who have sex with men. AIDS and Behavior. 2014; 18(2):254-262.

## Supplemental digital content C: Summary of characteristics of studies and effect sizes reporting on changes in sexual practices of men who have sex with men who became aware of HIV diagnosis in the United States

| Characteristics of Included Studies | Total Studies (n=20) | | |
| --- | --- | --- | --- |
|  | **Count*** | **%** | |
| Study Design |  |  | |
| Cross-sectional | 13 | 65 | |
| Pre-post | 7 | 35 | |
| Data Collection Start Year |  |  | |
| 1987-1996 | 4 | 20 | |
| 1997-2006 | 10 | 50 | |
| 2007-2016 | 6 | 30 | |
| Reported Outcomes |  |  | |
| Serosorting | 3 | 15 | |
| Seropositioning | 1 | 5 | |
| Outcome by partner type |  |  | |
| Partners at risk (HIV-uninfected or status unknown) | 11 | 55 | |
| Partner status unspecified | 18 | 90 | |
| Partner HIV-infected | 2 | 10 | |
| ART uptake by HIV-aware status reported | 7 | 35 | |
| Characteristics of Included Effect Sizes | **Total Effect Sizes (k=131)** | | |
|  | **Count** | | **%** |
| Outcome Type |  |  | |
| Categorical | 86 | 66 | |
| Continuous | 45 | 34 | |
| Outcome by Unit of Measurement |  |  | |
| Sexual dyad | 7 | 5 | |
| Last sex episode | 12 | 9 | |
| Any sex during recall period (1-96 months) | 112 | 86 | |
| Outcome by Partner Type |  |  | |
| Partners at risk | 76 | 58 | |
| Partner status unspecified | 47 | 36 | |
| Partner HIV-infected | 8 | 6 | |
| Follow-Up Interval: Months Since Diagnosis |  |  | |
| <6 | 12 | 9 | |
| 6-12 | 37 | 28 | |
| >12 | 32 | 24 | |
| NR | 50 | 38 | |
| Sero-adaptive Outcomes |  |  | |
| Serosorting | 5 | 4 | |
| Condom serosorting | 1 | 1 | |
| Oral sex serosorting | 1 | 1 | |
| Seropositioning | 2 | 2 | |
| Condom seropositioning | 2 | 2 | |
| Non-sero-adaptive Outcomes |  |  | |
| Occurrence of any sex | 1 | 1 | |
| Anal sex | 10 | 8 | |
| Number of sexual partners | 12 | 9 | |
| Partner serostatus irrespective of sexual act or condom use | 12 | 9 | |
| Unprotected sex overall | 9 | 7 | |
| Unprotected anal intercourse (UAI) | 37 | 28 | |
| Unprotected insertive anal intercourse (UIAI) | 26 | 20 | |
| Unprotected receptive anal intercourse (URAI) | 13 | 10 | |

*****Most papers report several types of outcomes, causing totals exceed 100%.

## Supplemental digital content D: Study designs and important risk of bias for included studies on changes in sexual practices of men who have sex with men who became aware of HIV diagnosis in the United States


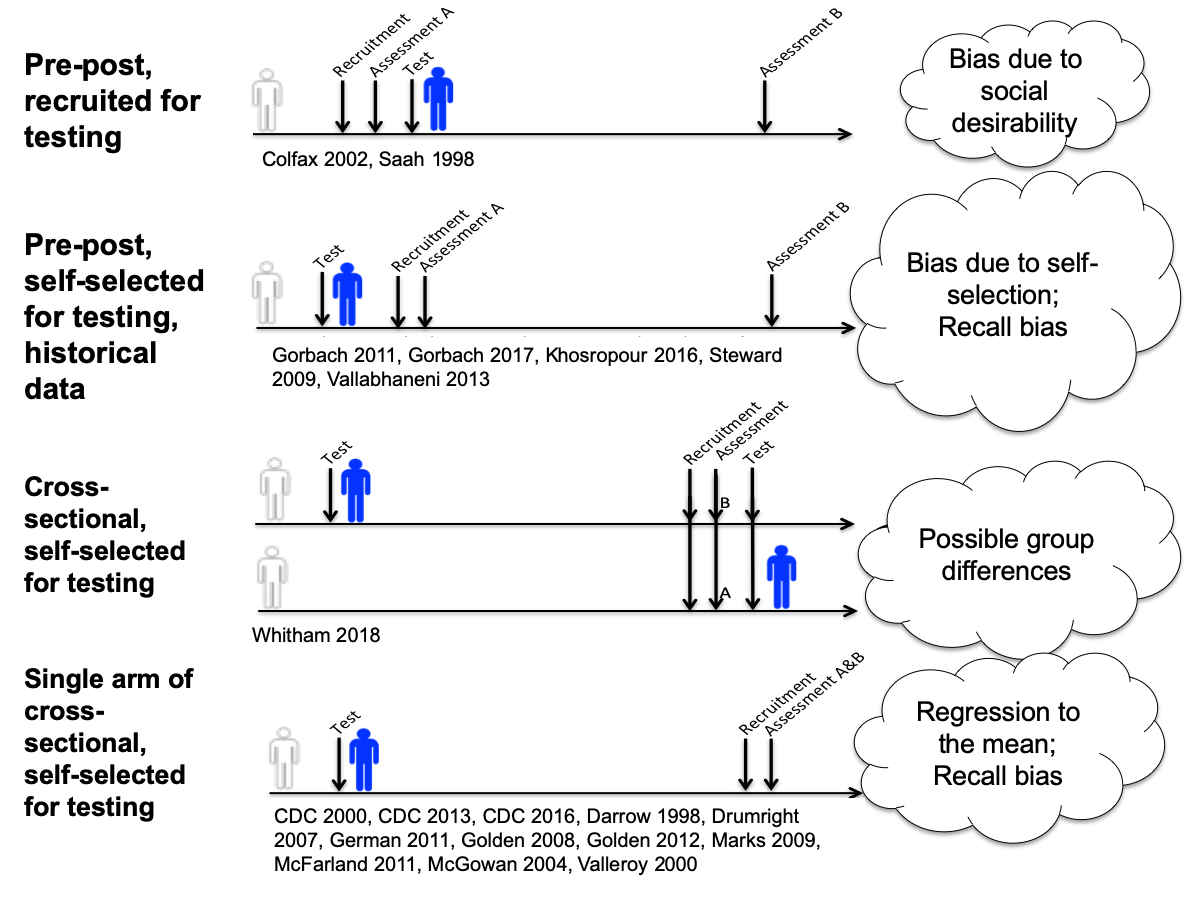


Unaware Aware


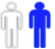


## Supplemental digital content E: Risk of bias among included studies on changes in sexual practices of men who have sex with men who became aware of HIV diagnosis in the United States

## Supplemental digital content F: Risk of bias of included studies on changes in sexual practices of men who have sex with men who became aware of HIV diagnosis in the United States

| **Author & Year** | **Incomplete outcome data, description of exclusions & attrition** | | **Selective outcome reporting** | **Failure to develop and apply appropriate eligibility criteria** | **Flawed measurement of exposure and/or outcome** | **Failure to control for confounders** | | | | | **Too-short or incomplete length of follow-up** | **Other bias** | |
| --- | --- | --- | --- | --- | --- | --- | --- | --- | --- | --- | --- | --- | --- |
| CDC 2000 | High | | Unclear | Low | High | High | | | | | Low | High | |
| CDC 2013 | Unclear | | Unclear | Low | High | High* | | | Low^†^ | | Low | Low | |
| CDC 2016 | Low | | Low | Low | High | High | | | | | Low | Unclear^‡^ | Low^§^ |
| Colfax 2002 | Unclear | | Low | Low | High | Low | | | | | Low | High | |
| Darrow 1998 | Low | | Unclear | Low | High | High | | | | | Unclear | Low | |
| Drumright 2007 | Low | | Low | Low | High | Unclear | | | | | Low | High | |
| German 2011 | Unclear^‖^ | Low^¶^ | Low | Low | High | High | | | | | Low | Low | |
| Golden 2008 | Low | | Unclear | Low | High | High | | | | | Low | Low | |
| Golden 2012 | Low | | Low | Low | High | High | | | | | Low | Low | |
| Gorbach 2011 | Unclear | | Unclear | Low | High | Low | | | | | Low | High | |
| Gorbach 2017 | High | | Low | Low | High | High | | | | | Low | High | |
| Khosropour 2016 | High | | Unclear | Low | High | Low | | | | | Low | High | |
| Marks 2009 | Unclear | | Unclear | Low | High | High | | | | | Low | Unclear | |
| McFarland 2011 | Unclear | | Unclear | Low | High | High | | | | | Low | Unclear | |
| McGowan 2004 | Low | | Low | Low | High | High | | | | | Low | High | |
| Saah 1998 | Unclear | | Low | Low | High | Low | | | | | Low | Low | |
| Steward 2009 | Unclear | | Unclear | Low | High | High** | Un-clear^††^ | | | Low^‡‡^ | Low | Unclear | |
| Vallabhaneni 2013 | Unclear | | Unclear | Low | High | Unclear^§§^ | | Low^‖‖^ | | | Low | High | |
| Valleroy 2000 | Unclear | | Unclear | Low | High | High | | | | | Low | High | |
| Whitham 2018 | Low | | Low | Low | High | High | | | | | Low | Unclear | |

*Cycle 2

^†^Cycle 3

^‡^ Any condomless anal sex (CAS), number of partners

^§^ Discordant CAS, CAS or oral sex with a casual male partner

^‖^ Wave 1

^¶^ Wave 2

**Percentage of partners who were HIV-infected, percentage of CAS acts with an HIV-infected partner

^††^ Number of partners per week

^‡^ Percentage of condomless sex acts

^§§^ 48-96 months

^‖‖^ 6-24 months
